# Effects of transgenic *Bacillus thuringiensis* cotton on insecticide use, heliothine counts, plant damage, and cotton yield: A meta-analysis, 1996-2015

**DOI:** 10.1371/journal.pone.0200131

**Published:** 2018-07-19

**Authors:** Daniel Fleming, Fred Musser, Dominic Reisig, Jeremy Greene, Sally Taylor, Megha Parajulee, Gus Lorenz, Angus Catchot, Jeffrey Gore, David Kerns, Scott Stewart, Deborah Boykin, Michael Caprio, Nathan Little

**Affiliations:** 1 Mississippi State University, Department of Biochemistry, Molecular Biology, Entomology, and Plant Pathology, Mississippi State, MS, United States of America; 2 North Carolina State University, Vernon G. James Research and Extension Center, Plymouth, NC, United States of America; 3 Clemson University, Edisto Research and Education Center, Blackville, SC, United States of America; 4 Virginia Tech, Tidewater Agricultural Research and Extension Center, Suffolk, VA, United States of America; 5 Texas A&M University, AgriLife Research and Extension Center, Lubbock, TX, United States of America; 6 University of Arkansas Cooperative Extension Service, Lonoke Extension Center, Lonoke, AR, United States of America; 7 Mississippi State University Delta Research and Extension Center, Stoneville, MS, United States of America; 8 Texas A&M University Department of Entomology, College Station, TX, United States of America; 9 The University of Tennessee, West Tennessee Research and Education Center, Jackson, TN, United States of America; 10 United States Department of Agriculture–Agricultural Research Service, James Whitten Delta States Research Center, Stoneville, MS, United States of America; 11 United States Department of Agriculture–Agricultural Research Service, Southern Insect Management Research Unit, Stoneville, MS, United States of America; Chinese Academy of Agricultural Sciences Institute of Plant Protection, CHINA

## Abstract

The primary management tactic for lepidopteran pests of cotton in the United States of America (USA) is the use of transgenic cotton that produces *Bacillus thuringiensis* Berliner (*Bt*) toxins. The primary target pests of this technology are *Helicoverpa zea* (Boddie) and *Heliothis virescens* (F.) in the eastern and central Cotton Belt of the USA. Concerns over the evolution of resistance in *H*. *zea* to *Bt* toxins and scrutiny of the necessity of *Bt* crops has escalated. We reviewed published and unpublished data from field trials of *Bt* cotton in the eastern and central Cotton Belt of the USA through 2015 to evaluate the effectiveness of *Bt* cotton (Bollgard, Bollgard II, WideStrike, WideStrike 3, and TwinLink). *Bt* cotton reduced insecticide usage, reduced heliothine pest numbers and damage, and provided a yield benefit, but Bollgard II and WideStrike efficacy declined in the Midsouth over the period evaluated. In the Southeastern region, heliothine damage remained constant through 2015, but yield benefits declined from 2010 until 2015. Resistance of *H*. *zea* to several *Bt* toxins is the most plausible explanation for the observed changes in *Bt* cotton efficacy. The introduction of new *Bt* toxins such as found in Widestrike 3 and Twinlink may preserve the benefits of *Bt* crops. However, while both Widestrike 3 and Twinlink had less damage than Widestrike, damage levels of both were similar to Bollgard II.

## Introduction

### *Bt* crops

**Fig 1:**
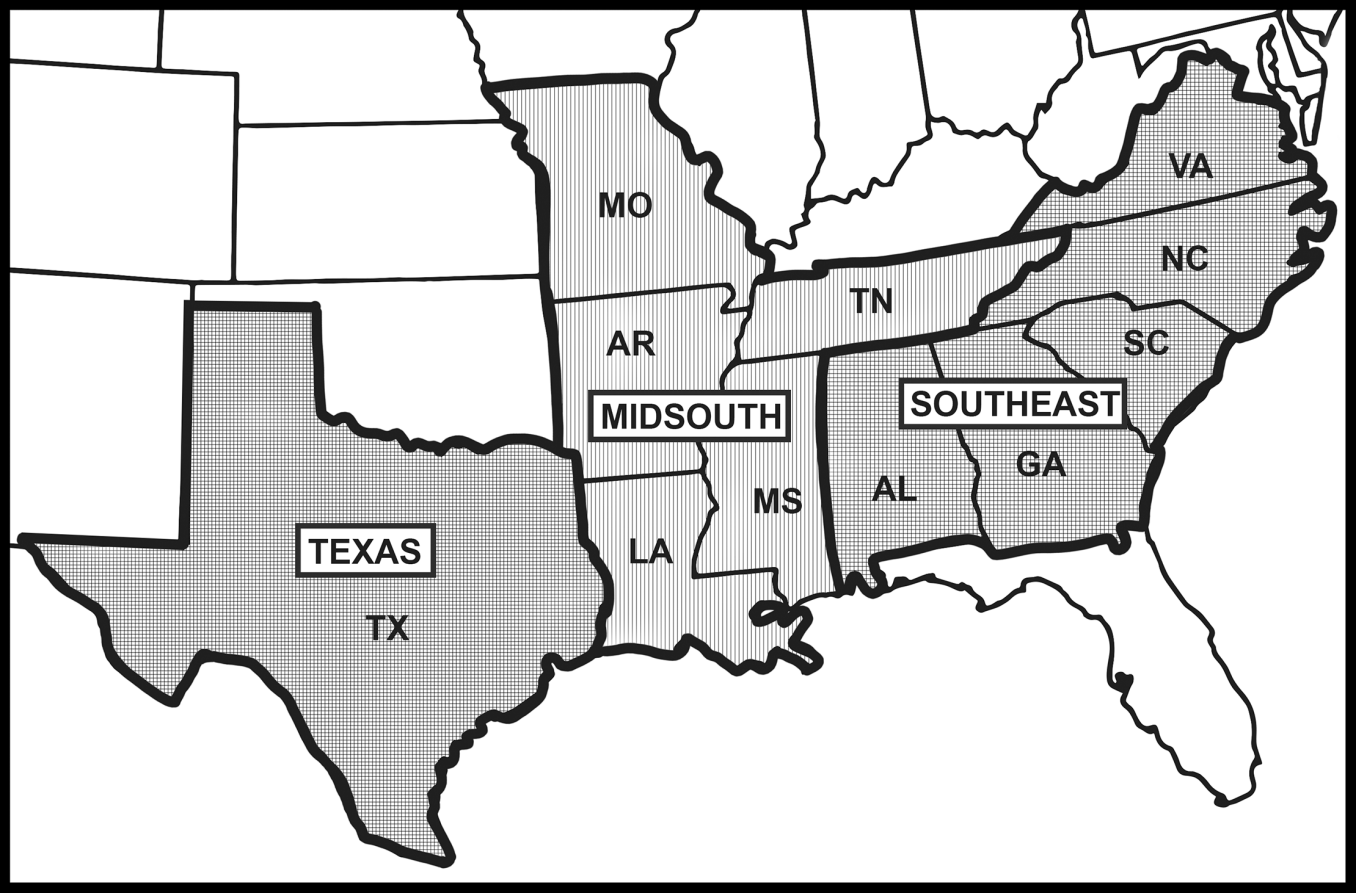
Map of the eastern and central Cotton Belt of the United States indicating the regions and states of trial locations used for analyses in this paper.

Lepidopteran insect control in transgenic crops is accomplished through the insertion of genes from the bacterium *Bacillus thuringiensis* Berliner (*Bt*). These genes encode for proteins with insecticidal activity in the midgut of targeted insect species. Five types of transgenic *Bt* cotton (*Gossypium hirsutum* L.) were commercialized between 1996 and 2015 in the United States (Table 1). In 2015, there were approximately 3.1 million hectares of cotton grown in Texas, the Midsouth and the Southeast combined (Fig 1), with transgenic *Bt* cotton planted on approximately 2.2 million hectares [1].

**Table 1:** Cotton technologies with transgenes from *Bacillus thuringiensis* Berliner (*Bt*) commercialized in the United States, 1996–2015.

Technology	Year of commercial availability	*Bt* transgene(s)	Event
**Bollgard**	1996	Cry1Ac	Mon531
**Bollgard II**	2003	Cry1Ac, Cry2Ab	Mon15985
**WideStrike**	2005	Cry1Ac, Cry1F	3006-210-23 + 281-24-236
**TwinLink**	2014	Cry1Ab, Cry2Ae	T304-40 + GHB119
**WideStrike 3**	2015	Cry1Ac, Cry1F, Vip3A	3006-210-23 + 281-24-236 + Cot102

**Fig 2:**
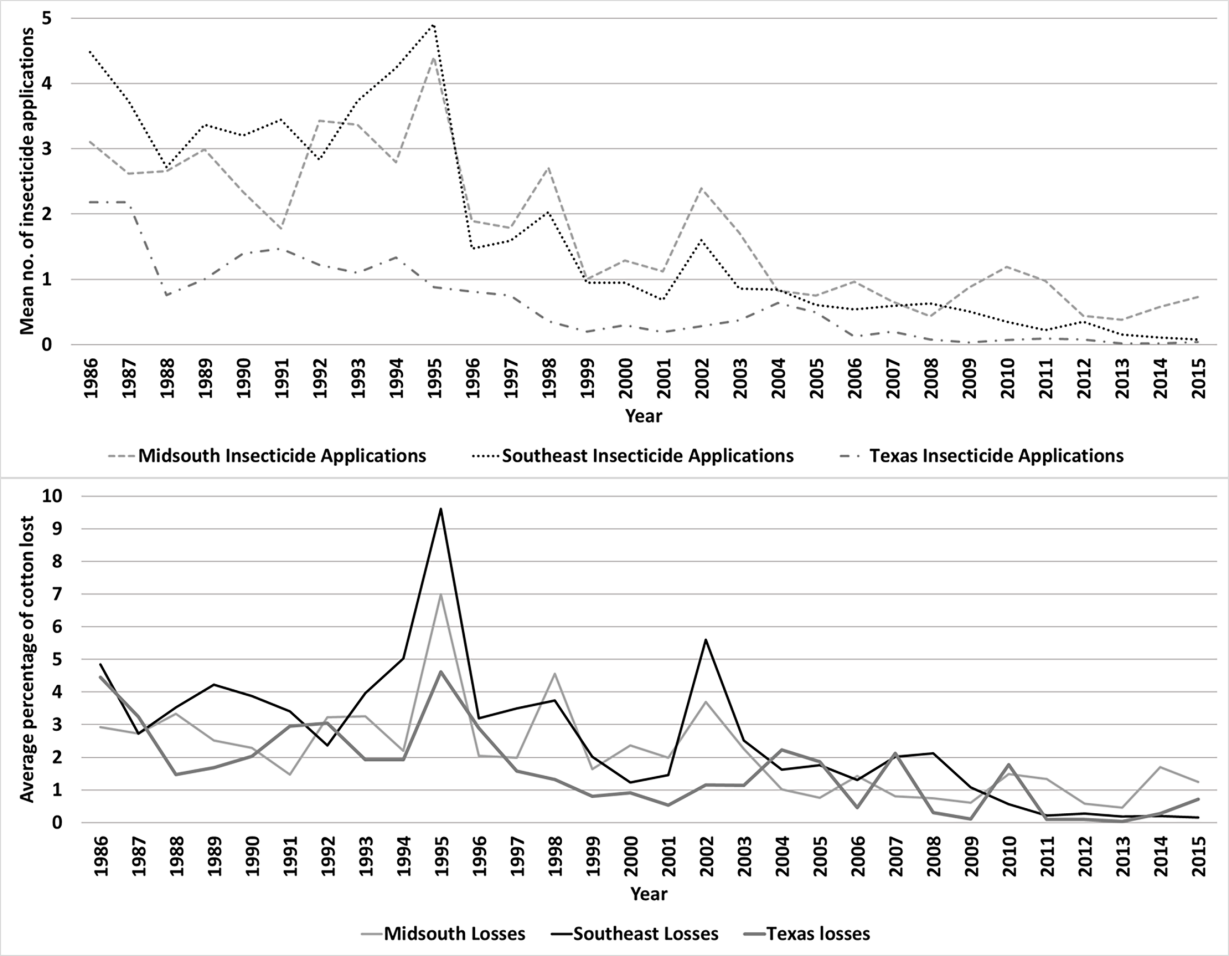
Changes in insecticide applications and yield losses in cotton due to heliothine infestations in the eastern Cotton Belt of the United States, 1986–2015. Compiled from Williams [1].

The primary pests targeted for control with *Bt* cotton in these regions are the heliothine species *Helicoverpa zea* (Boddie) (bollworm, corn earworm) and *Heliothis virescens* (F.) (tobacco budworm). These pests damage cotton by feeding primarily on and within the fruiting structures. Newly hatched *H*. *zea* and *H*. *virescens* larvae feed on plant terminals, then move to small squares, then larger squares, then bolls [2]. Estimates of insecticide usage and damage losses associated with these species following the introduction of *Bt* cotton (data from 1986–1995 compared to 1996–2015) were reduced by 61% and 47%, 79% and 60%, and 81% and 63%, respectively, in the Midsouth, Southeast, and Texas, respectively. [1] (Fig 2).

Many of the same *Bt* genes have been introduced into corn to control various lepidopteran pests, including *H*. *zea*. This technology has been widely accepted by corn growers, grown on 81% of the area planted to corn in the U.S. in 2015 [3]. *Bt* corn was also commercially introduced in 1996, so exposure to the *Bt* toxins in both crops has occurred simultaneously.

*Helicoverpa zea* is a pest of both cotton and corn, and populations of *H*. *zea* may spend as many as four generations per year in these crops [4–6]. Populations occurring in areas where *Bt* corn and cotton are both grown are potentially exposed to the Cry1A, Cry1F, Cry2A, and Vip3A toxins in both crops. Corn is grown on approximately 3.4 million hectares in the eastern and central Cotton Belt, and the Environmental Protection Agency (EPA) mandates a planted refuge of non-*Bt* corn consisting of 50% or 20% of corn acres in cotton growing regions for single and multi-gene *Bt* corn varieties, respectively [7]. These refuge requirements are in place to slow resistance of pests to the *Bt* toxins; however, as few as 40 percent of growers adhere to the refuge requirements [8, 9], potentially resulting in the production of fewer susceptible individuals than desired for resistance management.

### Concerns over resistance to *Bt* technology

Simulation models indicated that *H*. *zea* resistance to single-gene *Bt* crops could occur within 7 to 30 years [5, 10–13], while dual-gene crops would be expected to last longer [13]. The pyramiding of multiple toxins and a refuge strategy were implemented to slow the development of resistance of the major target pests to *Bt* crops [14–18]. Thus far, field-evolved *Bt* resistance has not been documented for *H*. *virescens*; however, laboratory selection of a Cry1Ac resistant colony has occurred [19]. Field-evolved resistance in populations of *H*. *zea* has been documented for Cry1Ab, Cry1Ac, and Cry1A.105+ Cry2Ab toxins in several locations [20–24].

Several factors may be solely or cumulatively responsible for *H*. *zea* resistance, including exposure of multiple generations of *H*. *zea* per year to *Bt* toxins in corn and cotton, lack of compliance with EPA mandated refuge requirements, exposure to the same *Bt* genes for many years, cross resistance to multiple *Bt* toxins, and the failure to express *Bt* at a high-dose from the outset [18]. Cry1Ab and Cry1Ac genes were the first *Bt* toxins commercially available and they are still found in most varieties of *Bt* corn and cotton after 20 years. The second *Bt* gene introduced for lepidopteran control in corn during 2001 and cotton during 2003 was Cry1F, and this gene also remains in many commercially available cotton and corn varieties. None of these toxins were ever considered to express a high-dose against *H*. *zea* [18, 25, 26]. Further increasing the likelihood of resistance development, various levels of cross-resistance to numerous Cry toxins has been documented in *H*. *zea* [11, 27, 28] as well as other Lepidoptera [26, 29, 30]. However, cross resistance to *Bt* toxins is not found in all studies [31]. Caprio [32] showed cross resistance has a negative impact on all resistance management strategies, but Caprio et al. [33] found that partial cross-resistance was of minor importance compared to refuge size in the evolution of resistance. The implications of continued exposure of *H*. *zea* to similar *Bt* toxins in multiple crops is not fully known, but all these studies suggest that declining efficacy of these toxins against *H*. *zea* should be expected.

### Need for a meta-analysis

Evaluations of *Bt* cotton efficacy on lepidopteran pests has typically involved laboratory experiments with meridic diet or plant expressed protein and insect colonies from rearing facilities. Only six refereed articles [34–39] involving replicated field experiments and natural heliothine populations in the USA have been published. These experiments are important because they validate laboratory research in biologically relevant situations, revealing the strengths and weaknesses of *Bt* cotton in a range of environments and pest densities. The use and benefits of *Bt* cotton is complex when considering the differences in environment, pest populations, and IPM strategies across the country, and as a result, data from field experiments are highly variable or “noisy” on an individual basis [40]. Compiling large numbers of experiments together in a meta-analysis increases the precision of estimation, allowing researchers to detect small changes in susceptibility or other variables that are not possible with individual experiments [41, 42].

Five of the published field studies evaluated Cry1Ac (Bollgard), three evaluated Cry1Ac + Cry2Ab2 (Bollgard II), and one evaluated Cry1F + Cry1Ac (WideStrike). All experiments occurred between 1998 and 2003. The findings of these papers showed that *Bt* cotton reduced lepidopteran populations and the damage they cause and that this reduction further improved with the introduction of dual-gene technology. It has been nine years since the last refereed paper was published, and over fourteen years since the experiment was conducted. Since then, two *Bt* cotton technologies with three *Bt* genes new to cotton have been made commercially available (Table 1). Reduced efficacy of the older, single-gene technology has not been empirically demonstrated in field trials, nor has the efficacy of the older dual-gene technologies (Bollgard II and WideStrike), and the new dual- (TwinLink) and triple-gene (WideStrike 3) technologies been compared across multiple cotton growing regions. The results of this study will be important in predicting the longevity and benefits of the recently commercialized TwinLink Plus and Bollgard 3 technologies.

### Objectives

Our primary objective is to summarize transgenic *Bt* cotton efficacy and yield data produced from 1996 to 2015 in field experiments that used natural heliothine populations in the USA. Trial locations ranged from Virginia to Texas, as these are the cotton production regions that frequently experience *H*. *zea* feeding. We used data from trials making threshold-based insecticide applications to assess the impacts of *Bt* technology on insecticide usage. Additionally, we used trials where insecticides targeting heliothines were not applied, to determine if changes in efficacy or yield have occurred over time and to compare efficacy and yield of various *Bt* and non-*Bt* varieties.

## Methods

### Compiling the dataset

**Fig 3:**
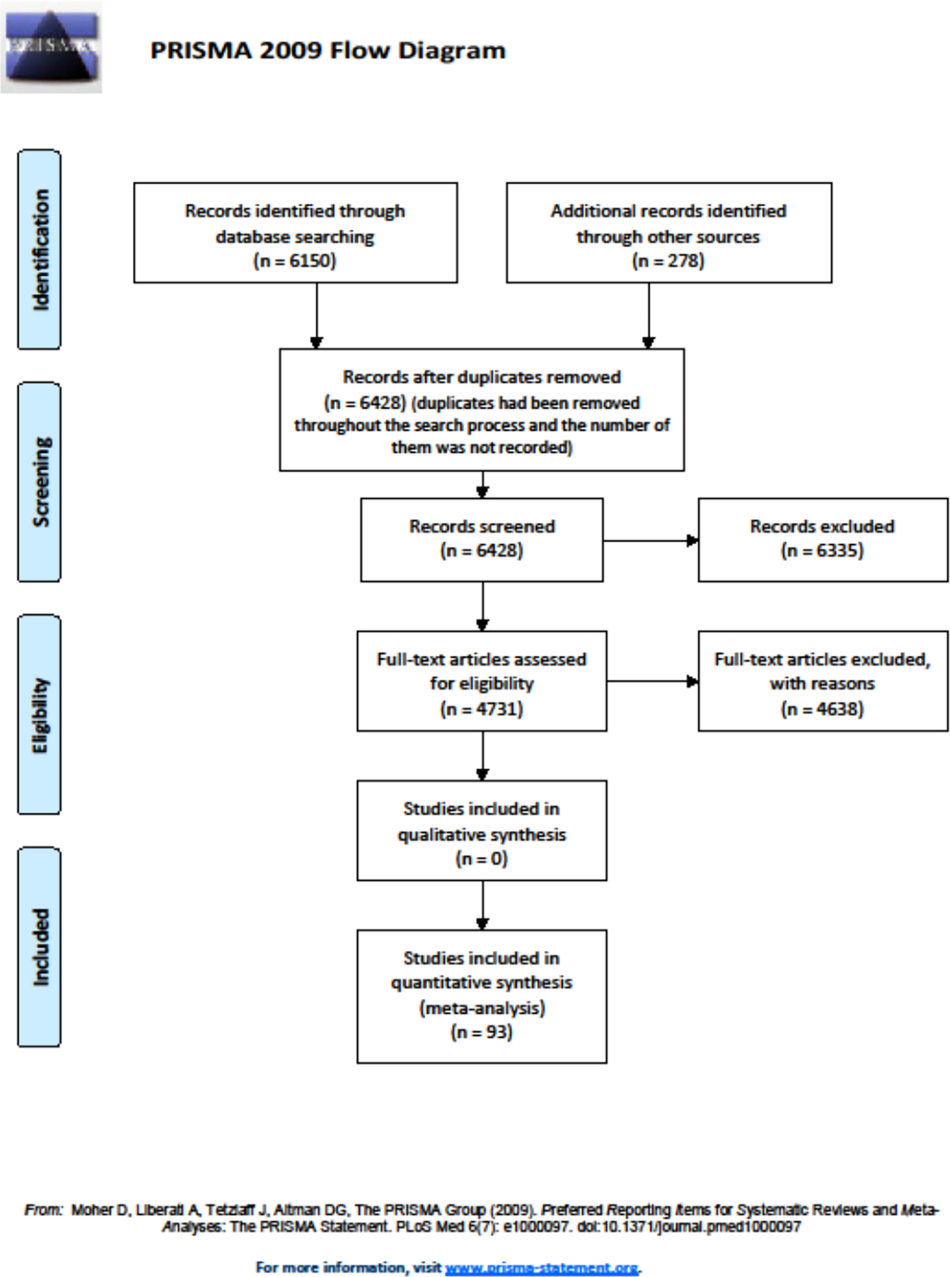
The PRISMA flow diagram[43].

Articles containing information on *Bt* cotton used in field experiments were identified using a combination of the terms *Bacillus thuringiensis*, *Gossypium hirsutum*, and one of the following: *Helicoverpa zea* or *Heliothis virescens*. Searches were conducted in Google Scholar, EBSCO through the Mississippi State University Library Discovery Service, Oxford University Press, Science Direct, Scopus, PubMed, BioOne, ISI Web of Knowledge, and the Proceedings of the Beltwide Cotton Conferences. Searches were limited to articles published no earlier than 1996. Article citations were imported into EndNote (v. X5.0.1, Thomson Reuters, www.endnote.com) and titles and abstracts were read to determine if the article contained data relevant to our objectives. Data were used if the trials included a non-*Bt* and a commercialized *Bt* variety, were conducted in a field setting, relied upon natural heliothine populations, provided a measure of variance, and if the number of observations could be determined from the information provided. Additional information was requested from authors if information in the article was insufficient or needed further clarification. In addition to these published articles, current university research and Extension Service entomologists working with cotton in the target regions were asked to provide unpublished data that met the same requirements. Researchers supplying unpublished data were asked for clarification of data they provided if information was lacking. Data that were still in doubt regarding their use in this study was ignored. All appropriate data were placed into a database for statistical analysis. While not a requirement, all but three sources of data used in the analysis were from university and private company sponsored research plots. Fig 3 shows the PRISMA Flow Diagram. The data used for meta-analysis can be found in the Mississippi State University Institutional Repository (http://hdl.handle.net/11668/14199). A Prisma checklist was included as supplemental information to the journal (S1 Fig) [43].

Data collected included the state, city and year of the research, the type and frequency of insecticide usage for heliothine pests, the plant part(s) evaluated, yield, type of evaluation (heliothine counts, plant damage, and cotton yield), mean values, number of observations, and a measure of variance. Insecticide application types were separated as blanket sprays (same insecticide, rate, and number of applications were used over both *Bt* and non-*Bt* varieties), threshold sprays (*Bt* and non-*Bt* varieties were treated independently as pests reached the threshold for each technology), or none (no insecticide was used to manage heliothines). The threshold used was based on larval density or fruit damage as recommended by the extension service where the trial was conducted. The *Bt* and non-*Bt* varieties were not necessarily genetically related but were varieties that had similar maturities and growth habits. The specific varieties compared are listed in the repository. The larvae of *H*. *zea* and *H*. *virescens* are difficult to distinguish in field settings [44, 45], therefore, very little species-specific information was available to allow our study to evaluate the effects of *Bt* technologies separately for these two species.

### Statistical analyses

The sources of reported data and numbers of observations for each technology were calculated in SAS Proc Tabulate (SAS Institute, Cary, NC, USA). Data from trials conducting threshold insecticide applications were used to evaluate the extent of insecticide reduction between *Bt* technologies (Bollgard, Bollgard II, and WideStrike; data for TwinLink and WideStrike 3 were insufficient for analysis) and non-*Bt* varieties. Differences in insecticide usage were calculated using the formula:

Number of applications for technology1—Number of applications for technology_2_ Differences were analyzed as paired t-tests (SAS Institute, Cary, NC, USA). Pairs were made whenever both technologies were tested within the same trial. For the remaining analyses, only data from trials not using foliar insecticide to manage heliothine pests were used.

Data evaluating heliothine counts, plant damage, and yield comparisons of *Bt* to non-*Bt* cotton included results from separate studies that varied over a wide range in values. Various metrics of effect size are used in meta-analysis in order to convert these measurements to a common scale. The log response ratio [46, 47] is recommended where the outcome expresses the magnitude of the response to an experimental treatment by comparing to an experimental control group. The log response ratio (RR) and the scaled sampling variance of this metric (V_RR_) are defined as follows:

$$\begin{array}{l} \mathrm{RR}=\ln\;\left(\left[\mathrm{Mea}n_{\mathrm{Bt}\;\mathrm{value}}+1\right]/\left[\mathrm{Mea}n_{\mathrm{non}‑\mathrm{Bt}\;\mathrm{value}}+1\right]\right)\\ V_{\mathrm{RR}}={\left(\mathrm{Standard}\;\mathrm{Erro}r_{\mathrm{Bt}\;\mathrm{value}}\right)}^{2}/{\left(\mathrm{Mea}n_{\mathrm{Bt}\;\mathrm{value}}+1\right)}^{2}+{\left(\mathrm{Standard}\;\mathrm{Erro}r_{\mathrm{non}‑\mathrm{Bt}\;\mathrm{value}}\right)}^{2}/{\left(\mathrm{Mea}n_{\mathrm{non}‑\mathrm{Bt}\;\mathrm{value}}+1\right)}^{2} \end{array}$$

We modified the original formulas to use Mean + 1 in place of a mean. In some cases, the mean was zero or close to zero which caused problems when dividing by zero or a very small number.

To estimate overall means for the log response ratio and detect what factors might affect this ratio, analysis of variance was performed using a general linear mixed model (PROC GLIMMIX, SAS Institute, Cary, NC USA). Data were initially analyzed without using any weighting method but this was rejected because the quality of the V_RR_ data available from some studies was much better than from other studies. This was generally not a reflection of sample size, but of the statistics available to estimate V_RR_. Secondly, the inverse V_RR_ weighting method [48] was tested. This weighting method was also rejected because weights varied by more than 1000 times in some comparisons, giving an excessive amount of weight to a small number of studies.

As a compromise between no weighting and the inverse V_RR_ weighting method, the V_RR_ were sorted from low to high and assigned a weight from 1 to 5 based on their rank. Those trials having the smallest 20% of V_RR_ were assigned a weight of 5. Those trials in the second lowest 20% were given a weight of 4 and so on, so that the 20% of observations with the largest V_RR_ were given a weight of 1. While we are not aware of this weighting system being used previously, it is basically a scaled version of the commonly used inverse V_RR_ weighting system so that no individual trial counts more than five times more than the poorest trial in the analysis.

As mentioned above, there were limited data available to estimate the V_RR_ of some trials. An estimate of variance was needed to calculate V_RR_ and these estimates were difficult to obtain from some studies. Variances were estimated for each *Bt*: non-*Bt* and *Bt*: *Bt* comparison by determining a standard error of the difference (SE diff) for each comparison. The SE diff for data using the least significant difference (LSD) values to estimate variance was calculated as LSD/t-value. The SE diff for data using standard deviation (SD) to estimate variance was calculated as SE diff = (([technology_1_ SD^2^] / [technology_1_ n]) + ([technology_2_ SD^2^] / [technology_2_ n]))^0.5^. The SE diff for data using standard error (SE) to estimate variance was calculated as SE diff = ([technology_1_ SE^2^] + [technology_2_ SE^2^])^0.5^.

Data from trials pre-dating commercial availability of each technology (Table 1) were excluded from analyses as any changes prior to commercialization would be due to agronomic factors, and not *Bt* toxin effectiveness. Overall differences in response to the technologies were evaluated (reported as overall intercept in S6 Table). In addition, the main effects evaluated for heliothine counts and damage were plant part, region, and year, and the main effects evaluated for yield were region and year. The interaction of year and plant part was evaluated for heliothine counts and plant damage, and the interaction of year and region was evaluated for heliothine counts, damage and yield. The three-way interaction of year, plant part, and region and the two-way interaction of plant part and region were not analyzed because of a lack of data. Analyses for all main effects were done independently (i.e. the impact of plant part was not tested in the same analysis as region) since the data that met the requirement for each analysis differed. For analyses, regions and plant parts not having at least five observations were excluded from analyses involving their respective effects. Year was analyzed as a continuous variable with linear and quadratic terms. The value of year was set as year of study—1995. To analyze year as a factor, there needed to be at least 3 observations for each of 5 years (but not necessarily consecutive years). This requirement meant that year could not be analyzed for WideStrike 3 and TwinLink as they had not yet been commercialized for 5 years by 2015. Years occurring at either end of the tested time scale with less than 3 observations were deleted. To test the interaction of region or plant part with year required a region or plant part to have at least five years of data with at least three observations per year. As a result, many of the year interactions included only two regions or plant parts due to insufficient data for one or more regions or plant parts. Least square means for technology comparisons were separated using Fisher’s Protected Least Significant Difference test (LSD) (α = 0.05). Significant regressions over time were simplified by removing the non-significant terms from the final equation. Data were tested for normality of distribution and examined for outliers more than three standard deviations from the predicted value. Nine comparisons were identified as outliers for one or more models. All outliers were for Bollgard to non-*Bt* or Bollgard 2 to non-*Bt* comparisons. All outliers occurred prior to 2009 and the *Bt* technology was always more effective than predicted by the model. Five of these outliers were from a single trial in Texas in 2004 when insect damage was high in the non-*Bt* plots, but no damage was observed in the Bollgard and Bollgard II plots. These outliers were deleted from the data set so that the analysis would not be skewed by these rare circumstances. The number of data points omitted was never more than 5% of the total number of data points analyzed for any comparison. Multiple regression was used initially for analysis, but due to a paucity of data in numerous areas, was not used because results of several factors were frequently driven by one or two trials.

## Results

### Literature review

Over 6,000 articles were examined for inclusion in this study. The articles (refereed or otherwise) used are listed in S1 Table. There were 910 comparisons of *Bt*: non-*Bt* cotton and 523 comparisons of *Bt* technologies to one another (S2 and S3 Tables). Additionally, 1,293 *Bt*: non-*Bt* comparisons and 915 comparisons of *Bt* technologies were collected from unpublished sources (S1–S3 Tables). Overall, 63%, 32% and 5% of the data were from the Midsouth, Southeast and Texas, respectively. No data for TwinLink or WideStrike 3 were available from Texas. The number of comparisons of *Bt*: non-*Bt* and *Bt*: *Bt* for heliothine counts, damage and cotton yield are given in S4 and S5 Tables.

### Threshold-based insecticide usage

Data from comparisons with insecticide targeting heliothines on a threshold basis were used to determine the extent of the reduction of insecticide usage resulting from using *Bt* cotton. Data from Bollgard, Bollgard II, and WideStrike were available. The use of these technologies reduced insecticide usage by 1.3 to 2.6 applications (Table 2) relative to non-*Bt* cotton. Bollgard II reduced insecticide usage by approximately 1.1 applications when compared to Bollgard and 0.8 applications when compared to WideStrike (Table 2).

**Table 2:** Paired t-test comparisons of insecticide applications based on larval thresholds for heliothine pests in trials in the eastern and central Cotton Belt of the United States for *Bt* and non-*Bt* cotton.

			Mean ± SE of the number of insecticide applications		Mean ± SE of the number of insecticide applications reduced			
				
						t-test results		
Technology 1	Technology 2	Study Years	Technology 1	Technology 2		df	t	p
Non-*Bt*	Bollgard	96–09	3.5 ± 0.2	2.1 ± 0.2	1.3 ± 0.1	61	9.7	<0.01
Non-*Bt*	Bollgard II	04–10	3.8 ± 0.5	1.3± 0.4	2.6 ± 0.4	17	7.3	<0.01
Non-*Bt*	WideStrike	06–11	3.5 ± 0.5	1.5 ± 0.4	2.0 ± 0.3	21	6.3	<0.01
Bollgard	Bollgard II	04–09	1.7 ± 0.7	0.6 ± 0.2	1.1 ± 0.4	8	2.5	0.04
WideStrike	Bollgard II	06–10	2.5 ± 0.5	1.6 ± 0.6	0.8 ± 0.3	12	3.2	<0.01

### Efficacy comparisons

**Fig 4:**
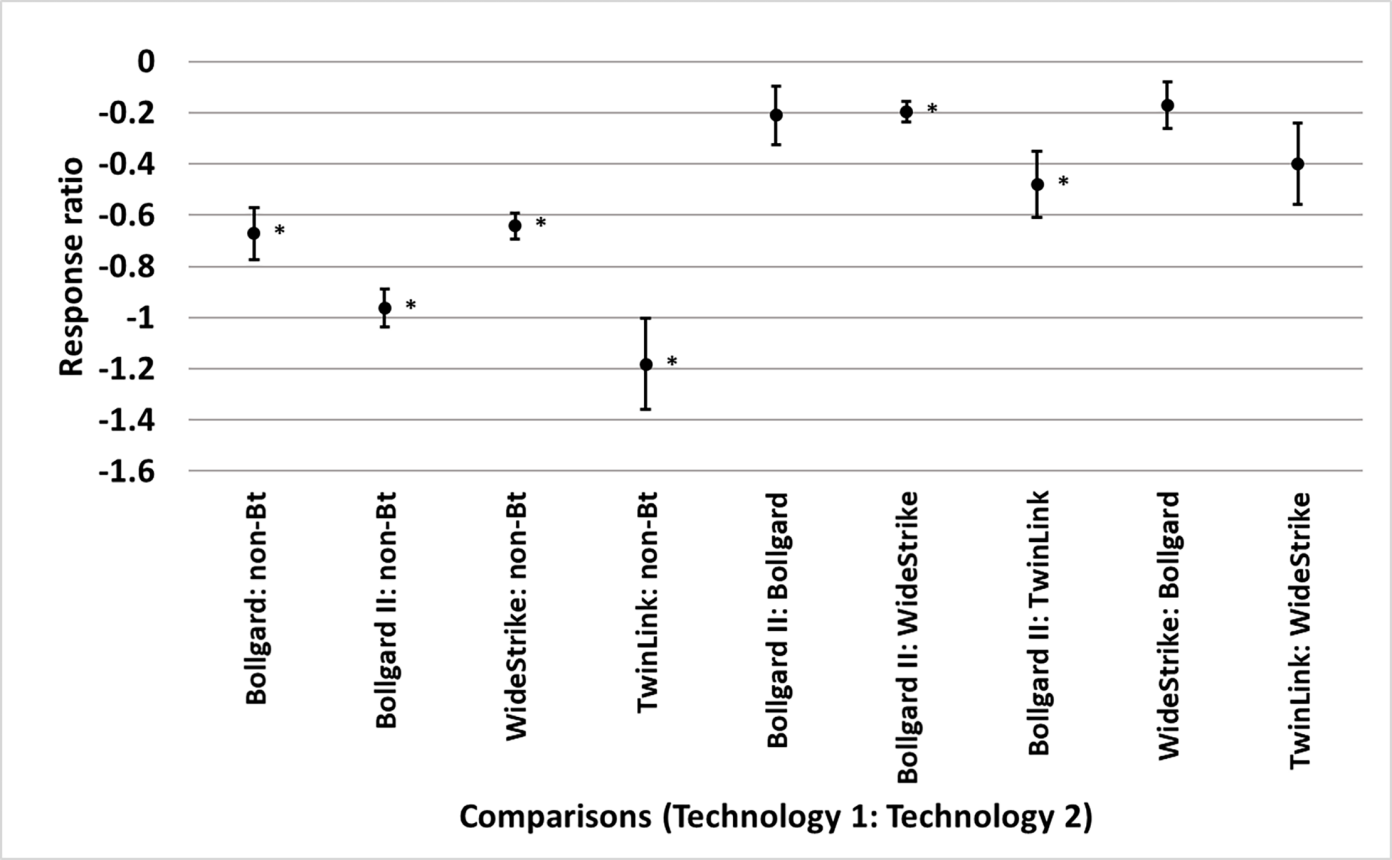
Least square mean ± SE of the response ratio of heliothine counts among comparisons of transgenic *Bacillus thuringiensis* (*Bt*) and non-*Bt* cotton in trials from the eastern and central Cotton Belt of the United States. Response ratio = ln ([Technology 1 mean_x_ + 1] / [Technology 2 mean_x_ + 1]). Comparisons marked by * indicate the technologies differed (t-test, p<0.05).

**Fig 5:**
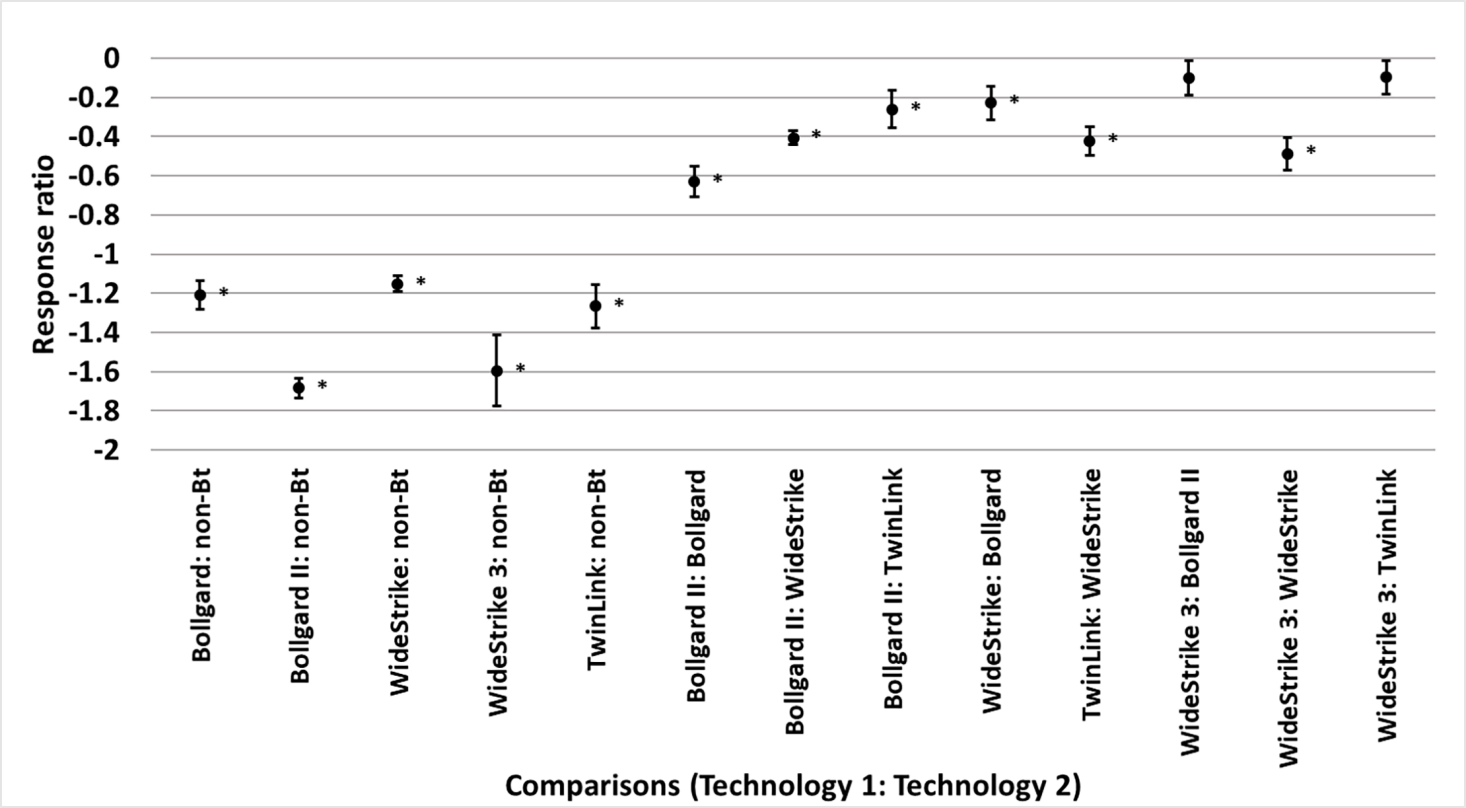
Least square mean ± SE of the response ratio of damage among comparisons of transgenic *Bacillus thuringiensis* (*Bt*) and non-*Bt* cotton in trials from the eastern and central Cotton Belt of the United States. Response ratio = ln ([Technology 1 mean_x_ + 1] / [Technology 2 mean_x_ + 1]). Comparisons marked by * indicate the technologies differed (t-test, p<0.05).

**Fig 6:**
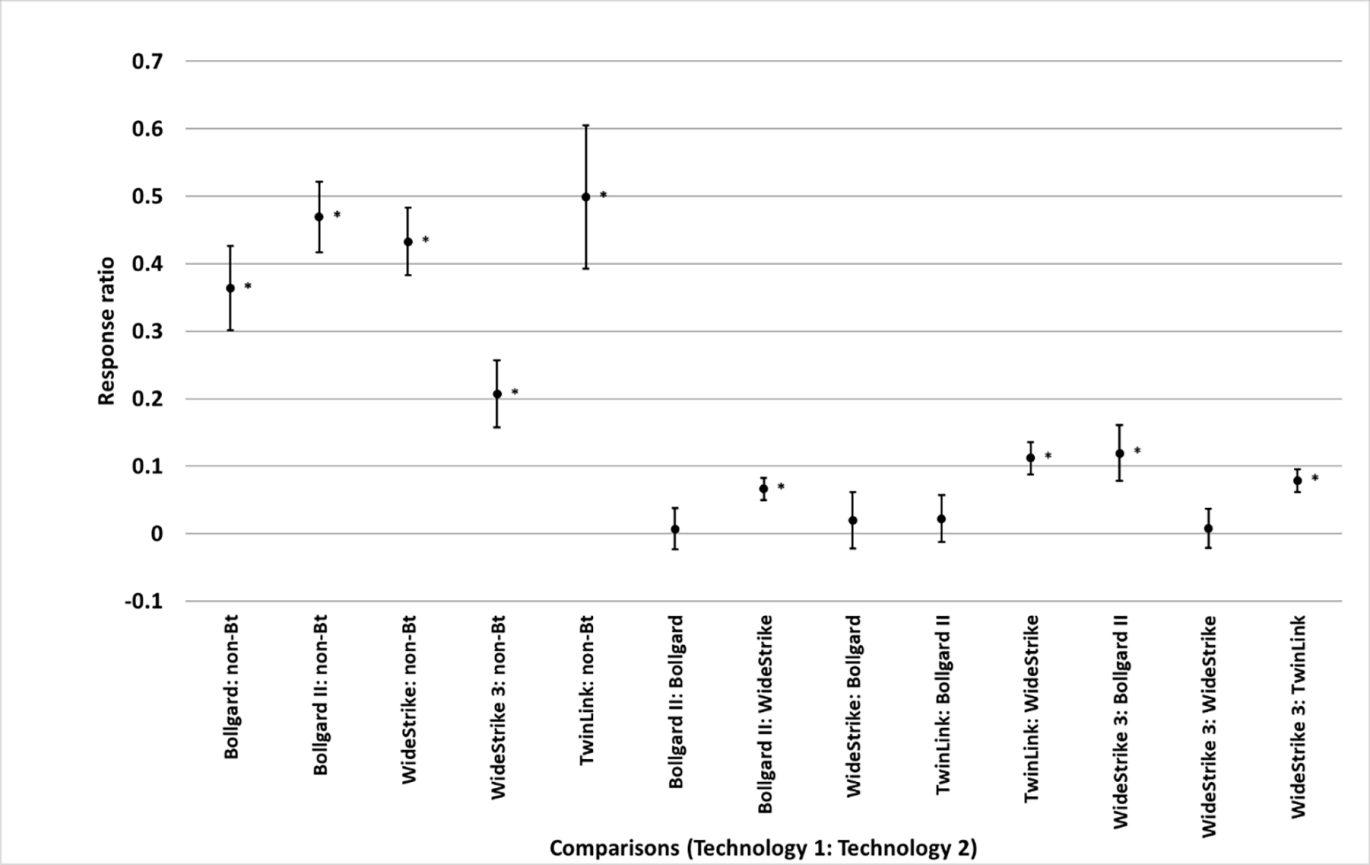
Least square mean ± SE of the response ratio of yield among comparisons of transgenic *Bacillus thuringiensis* (*Bt*) and non-*Bt* cotton in trials from the eastern and central Cotton Belt of the United States. Response ratio = ln ([Technology 1 mean_x_ +1] / [Technology 2 mean_x_ + 1]). Comparisons marked by * indicate the technologies differed (t-test, p<0.05).

Comparisons of *Bt* cotton to non-*Bt* and other *Bt* cotton types were conducted to determine the extent of reduction of heliothine counts and damage, how efficacy of *Bt* technologies compared to each other, and how yield was affected (S6 Table). Bollgard, Bollgard II, WideStrike, and TwinLink reduced heliothine infestations relative to non-*Bt* by 49% (p<0.0001), 61.8% (p<0.0001), 47.4% (p<0.0001), and 69.3% (p<0.0001), respectively. Bollgard II reduced heliothine infestations 17.9% more than WideStrike (p<0.0001) and 38.2% more than TwinLink (p = 0.004) (Fig 4). Bollgard, Bollgard II, WideStrike, WideStrike 3, and TwinLink reduced damage relative to non-*Bt* by 70%, 81%, 68%, 80%, and 72%, respectively (p<0.0001 for all technologies). Bollgard II reduced damage 47% more than Bollgard (p<0.0001), 33% more than WideStrike (p<0.0001), and 23% more than TwinLink (p = 0.010); TwinLink reduced damage 35% more than WideStrike (p<0.0001); WideStrike reduced damage 21% more than Bollgard (p = 0.015); and WideStrike 3 reduced damage 39% more than WideStrike (p<0.0001) (Fig 5). Bollgard, Bollgard 2, WideStrike, WideStrike 3, and TwinLink all improved yield relative to non-*Bt* by 44% (p<0.0001), 60% (p<0.0001), 54% (p<0.0001), 23% (p = 0.004), and 65% (p = 0.0002), respectively. Bollgard II and TwinLink had a higher yield than WideStrike of 7% (p = 0.0002) and 12% (p = 0.0003), respectively, and WideStrike 3 had a 13% higher yield than Bollgard II (p = 0.034) and 8% higher yield than TwinLink (p = 0.005) (Fig 6).

### *Bt* to non-*Bt* comparison: Effects of year, region, and plant part on heliothine counts and damage

**Fig 7:**
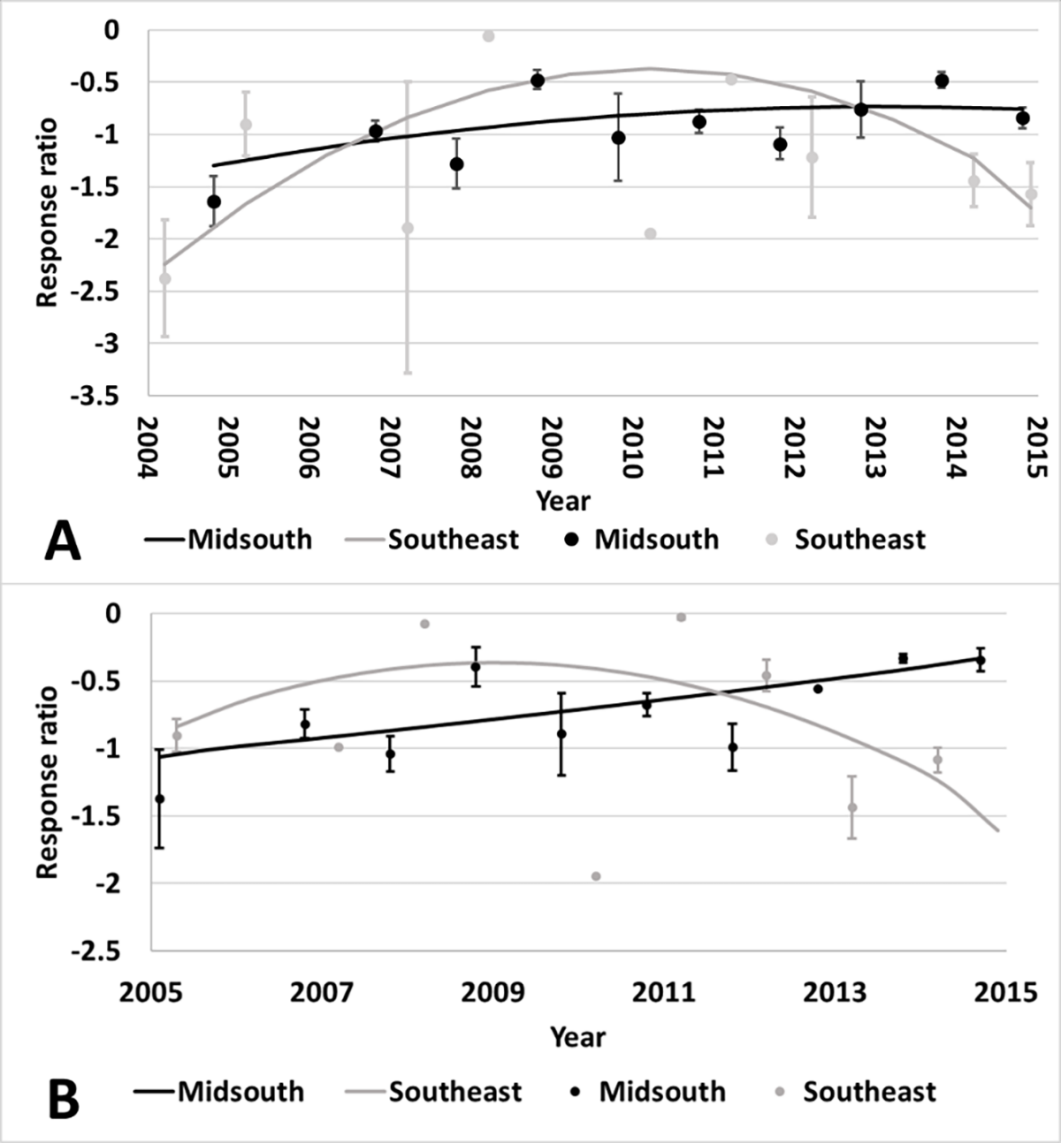
**Change over time of heliothine counts in Bollgard II (A) and WideStrike (B) cotton by region of the eastern and central Cotton Belt of the United States.** Bollgard II Midsouth equation: 0.0429x - 1.5111, Southeast equation: 1.569x - 0.05243x2–12.1179; WideStrike Midsouth equation: 0.0750x - 1.8438, Southeast equation: 0.9008x - 0.03258x2–6.5835. Response ratio (A) = ln ([Bollgard II mean_x_ + 1] / [non-*Bt* mean_x_ + 1]); Response ratio (B) = ln ([WideStrike mean_x_ + 1] / [non-*Bt* mean_x_ + 1]).

The main effects of year, region, and plant part and interactions of year with plant part and year with region were evaluated to determine if changes in *Bt* efficacy have occurred over time or if efficacy is different for plant parts or regions (S6 Table). There was an interaction of year and region for Bollgard II (p<0.01) and WideStrike (p<0.01) heliothine counts. The Midsouth had an increase in heliothine numbers collected from both Bollgard II and WideStrike relative to non-*Bt* as time progressed (Fig 7). Heliothine counts in the Southeast increased over time in Bollgard II and WideStrike relative to non-*Bt*; however, after 2010 counts began decreasing (Fig 7).

**Fig 8:**
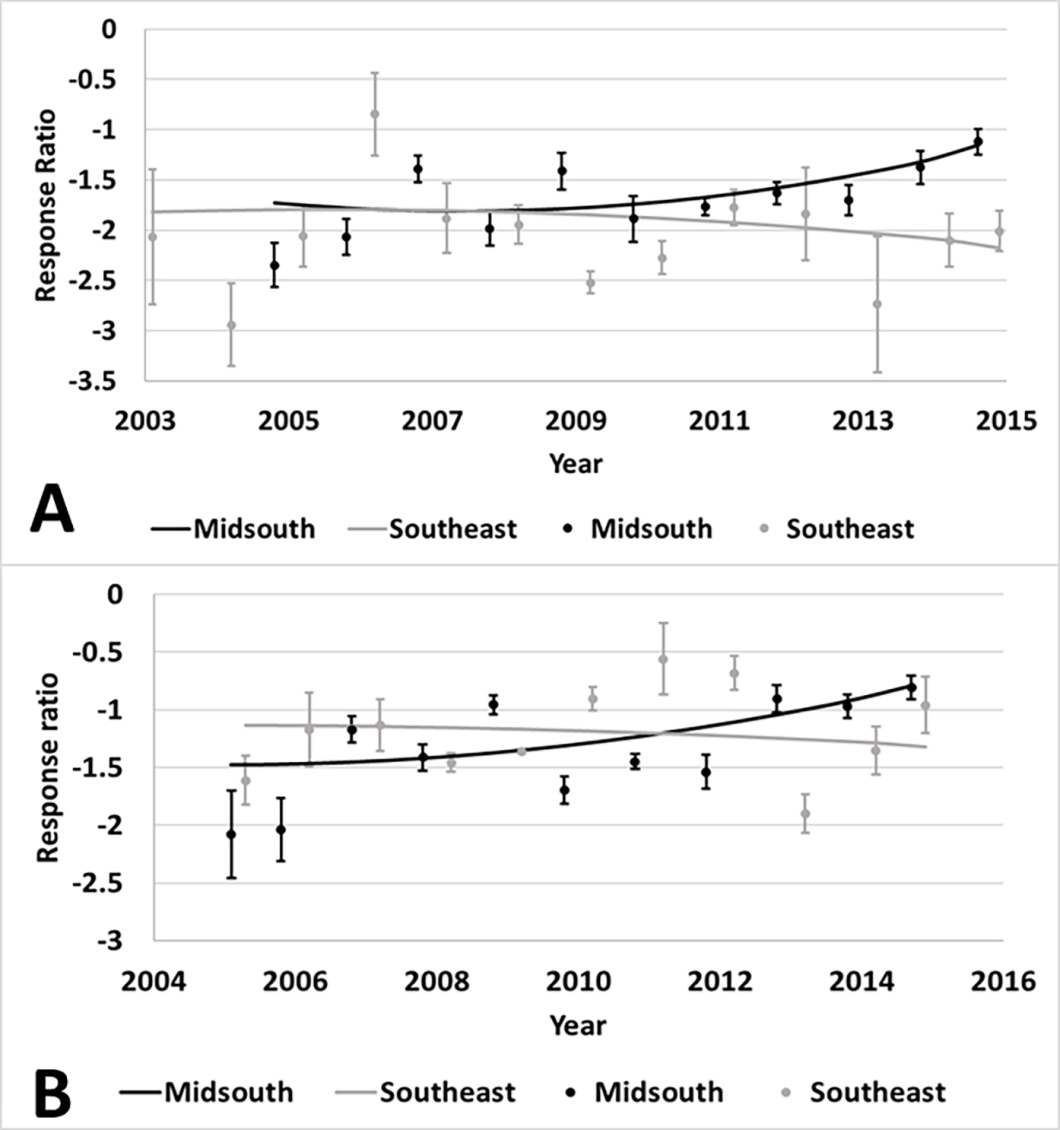
**Change over time of damage in Bollgard II (A) and WideStrike (B) cotton by region of the eastern and central Cotton Belt of the United States.** Bollgard II Midsouth equation: 0.0759x - 2.7923; Southeast equation: -1.9273; WideStrike Midsouth equation: 0.0776x – 2.4088; Southeast equation: -1.214. Response ratio (A) = ln ([Bollgard II mean_x_ + 1] / [non-*Bt* mean_x_ + 1]); Response ratio (B) = (ln([WideStrike mean_x_ + 1] / [non-*Bt* mean_x_ + 1]).

**Fig 9:**
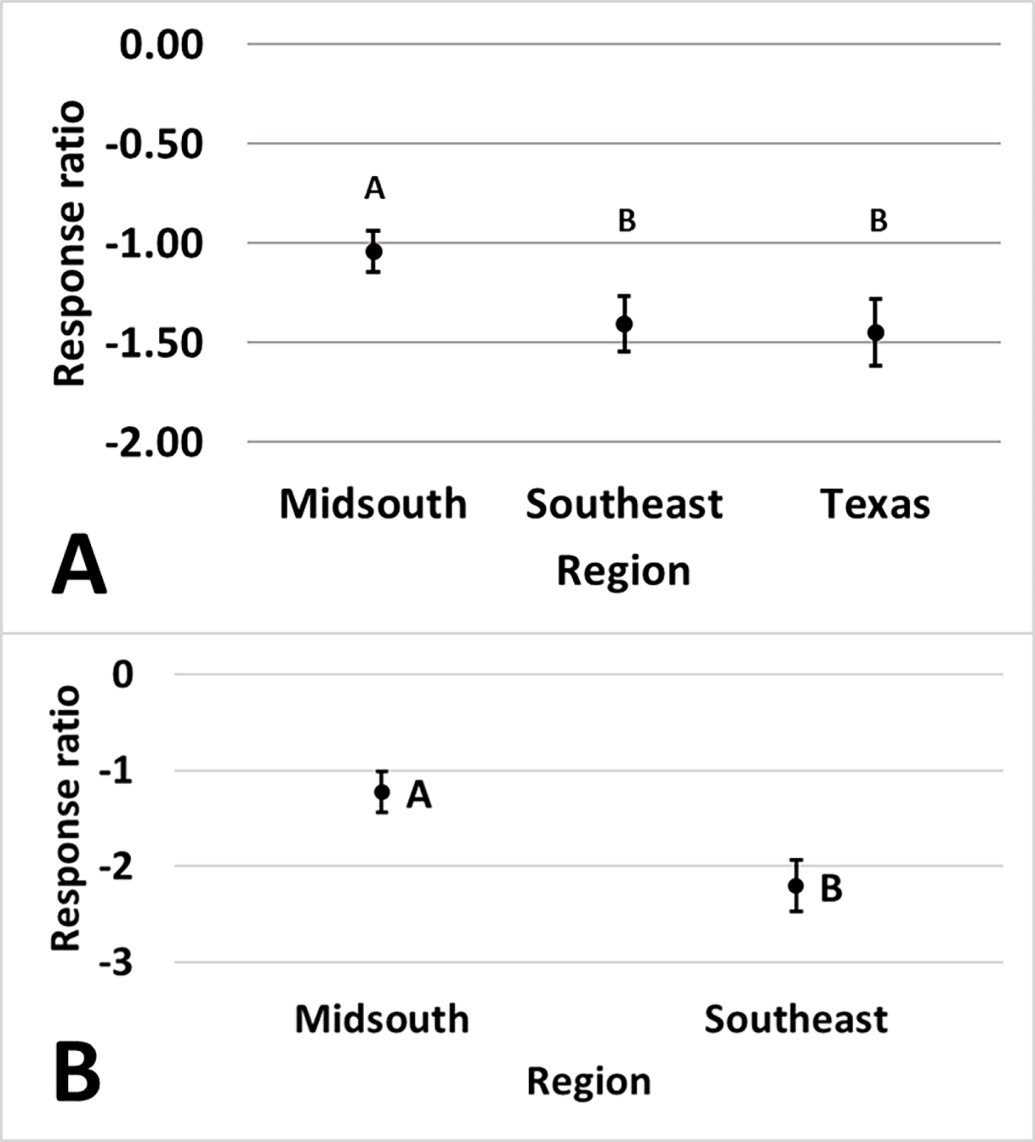
**Least square mean ± SE of the response ratio of region of Bollgard (A) and WideStrike 3 (B) damage data from trials in the eastern and central Cotton Belt of the United States.** Regions not sharing the same uppercase letter are different (Least square means α = 0.05). Response ratio (A) = ln ([Bollgard mean_x_ + 1] / [non-*Bt* mean_x_ + 1]); Response ratio (B) = ln ([WideStrike 3 mean_x_ + 1] / [non-*Bt* mean_x_ + 1]).

**Fig 10:**
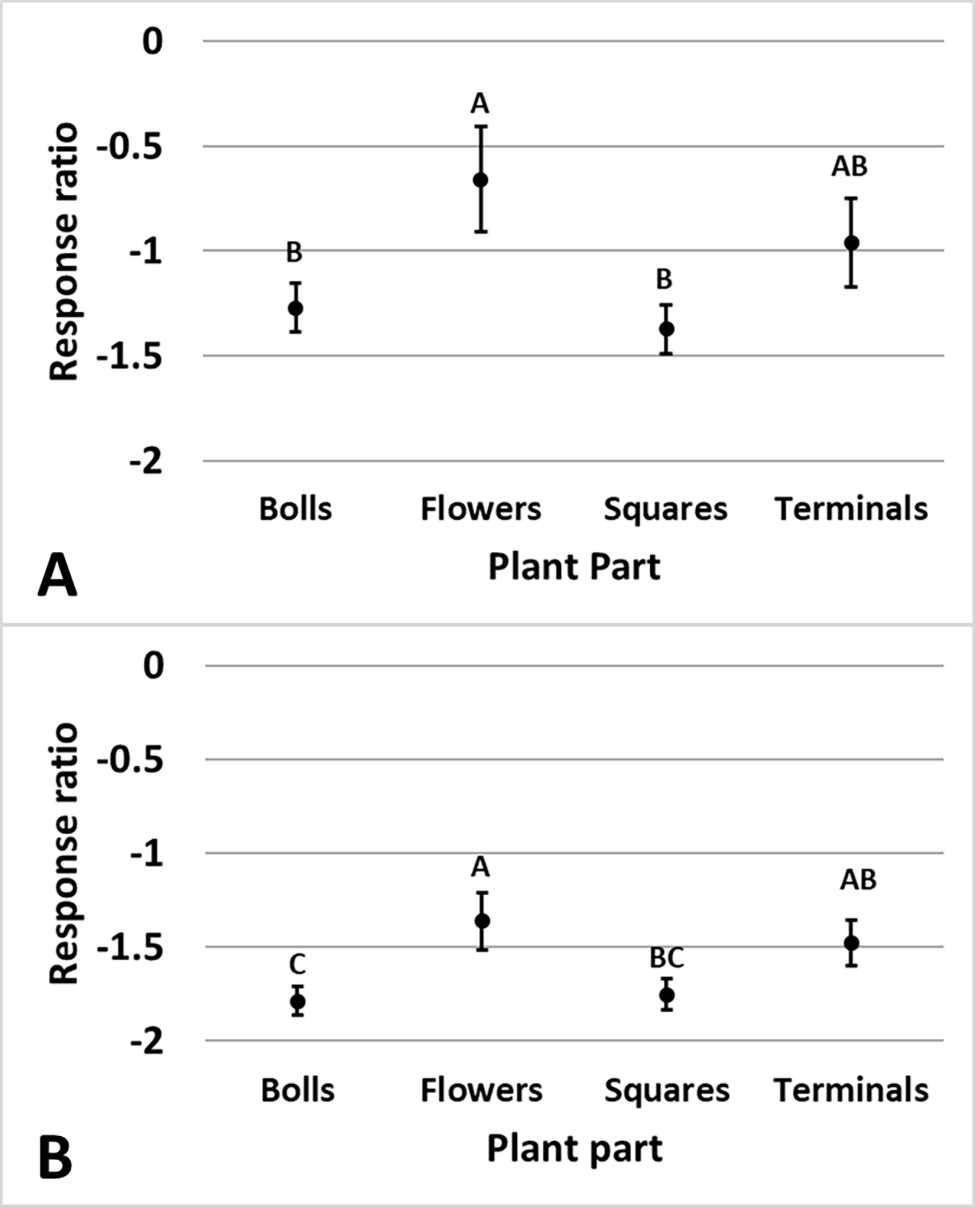
**Least square mean ± SE of the response ratio of plant part of Bollgard (A) and Bollgard II (B) damage data from trials in the eastern and central Cotton Belt of the United States.** Plant parts not sharing the same uppercase letter are different (Least square means α = 0.05). Response ratio (A) = ln ([Bollgard mean_x_ + 1] / [non-*Bt* mean_x_ + 1]); Response ratio (B) = ln ([Bollgard II mean_x_ + 1] / [non-*Bt* mean_x_ + 1]).

There was an interaction of year and region for Bollgard II (p<0.01) and WideStrike (p<0.01) damage. As time progressed, damage increased for both technologies in the Midsouth compared to non-*Bt*, but there was not a change in damage for either technology in the Southeast (Fig 8). Region influenced Bollgard (p = 0.040) and WideStrike 3 (p = 0.007) damage. Damage in Bollgard relative to non-*Bt* was reduced by 65% in the Midsouth compared to 74% and 77% in the Southeast and Texas, respectively (Fig 9). Damage in WideStrike 3 relative to non-*Bt* was reduced by 89% in the Southeast compared to 71% in the Midsouth. Plant part influenced the amount of damage reduction provided by Bollgard (p = 0.045) and Bollgard II (p = 0.022) technologies relative to non-*Bt*. Damage in Bollgard was reduced less on flowers (48%) than on bolls (72%) and squares (75%) (Fig 9). Damage in Bollgard II was reduced less on flowers (74%) than on bolls (83%) and squares (83%) and damage on terminals (77%) was reduced less than damage on bolls (83%) (Fig 10).

### *Bt* to non-*Bt* comparisons: Effects of year and region on yield

**Fig 11:**
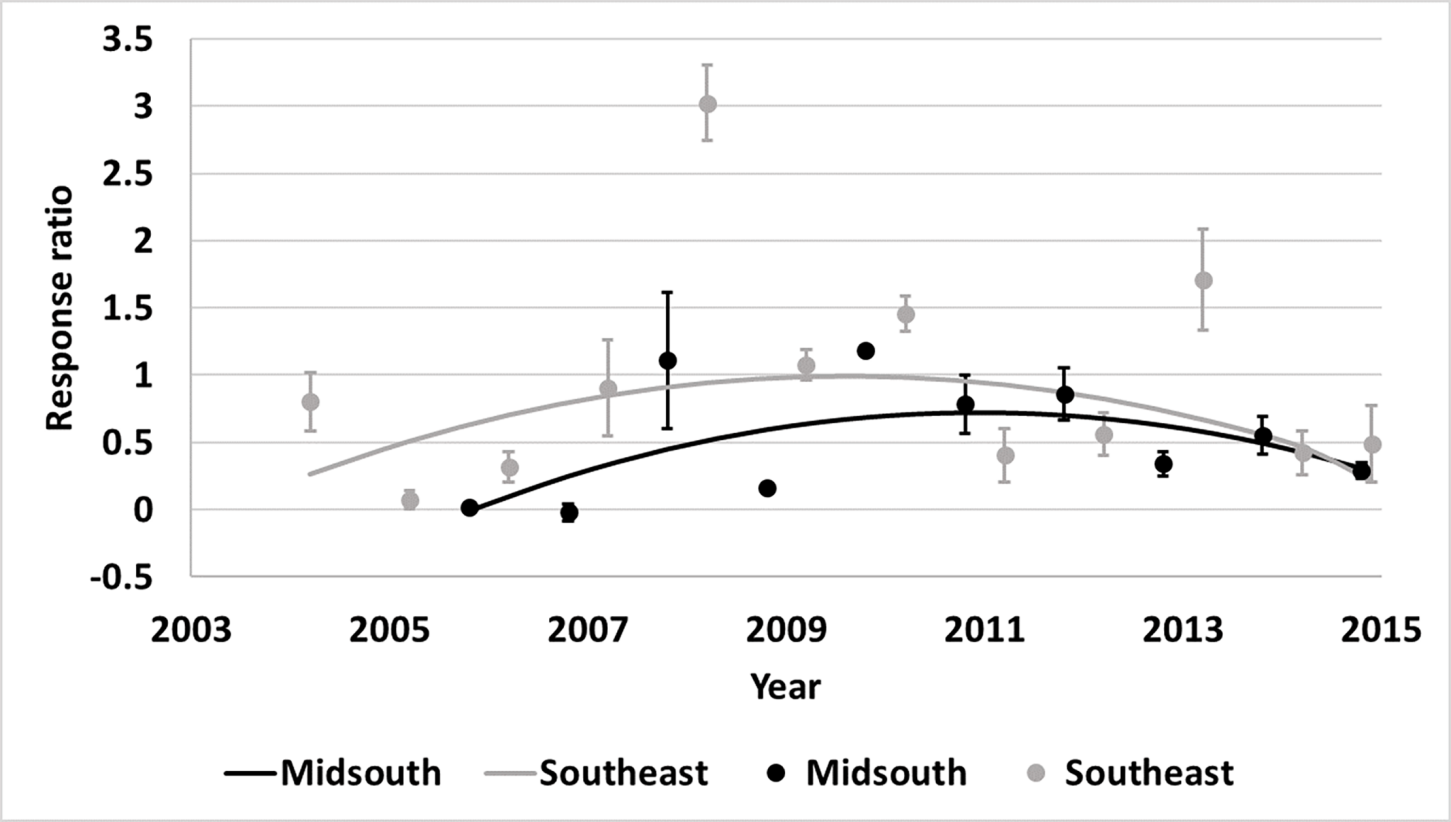
Change over time of yield in Bollgard II cotton by region in trials from the eastern and central Cotton Belt of the United States. Midsouth equation: 0.8941x - 0.02767x2–6.4911; Southeast equation: 0.7171x - 0.02489x2–4.1691. Response ratio = ln ([Bollgard II mean_x_ + 1] / [non-*Bt* mean_x_ + 1]).

**Fig 12:**
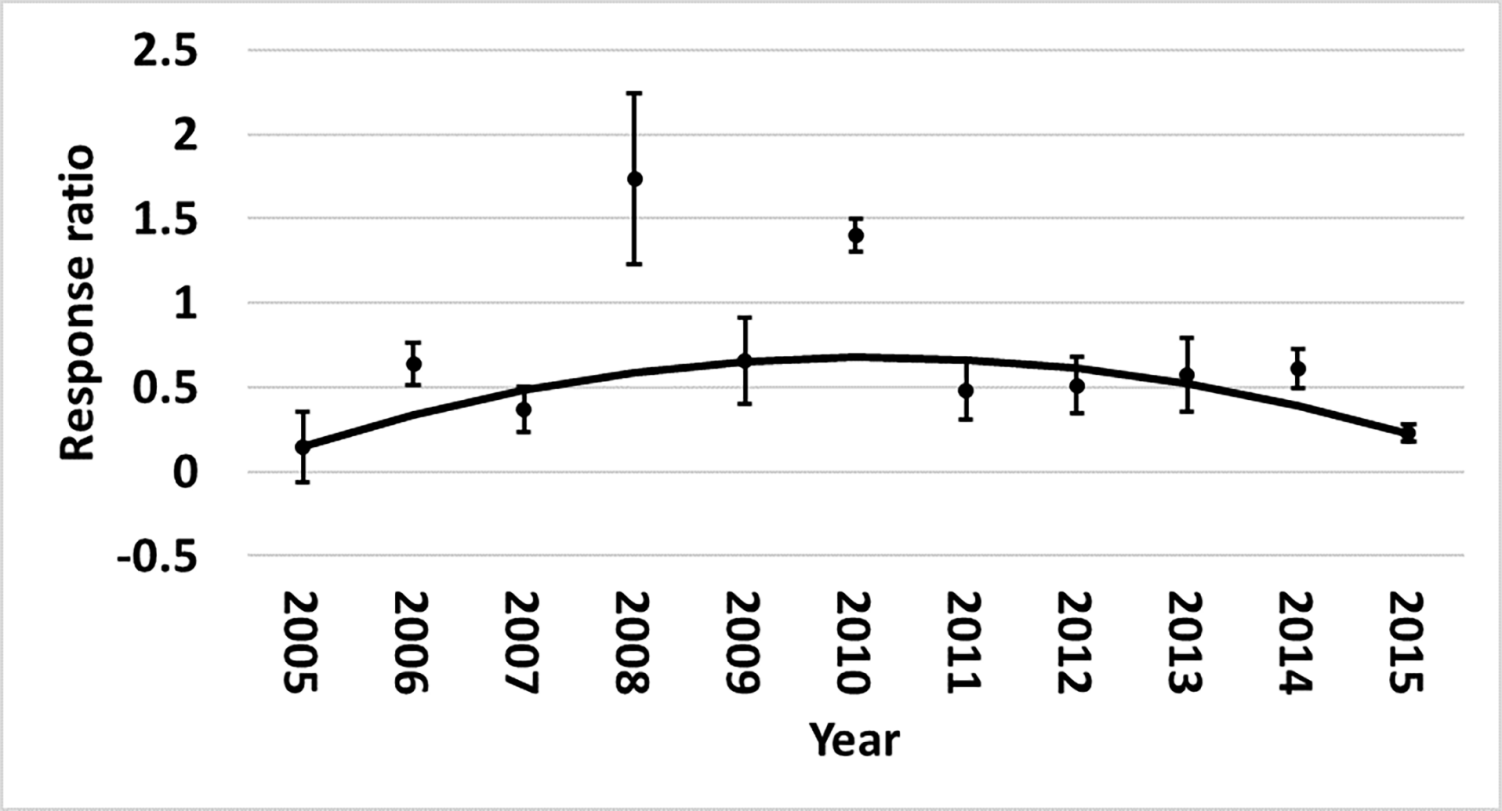
Change over time of yield in WideStrike cotton in trials from the eastern and central Cotton Belt of the United States. Equation: 0.593x - 0.01954x2–3.8209. Response ratio = ln ([WideStrike mean_x_ + 1 ] / [non-*Bt* mean_x_ + 1]).

**Fig 13:**
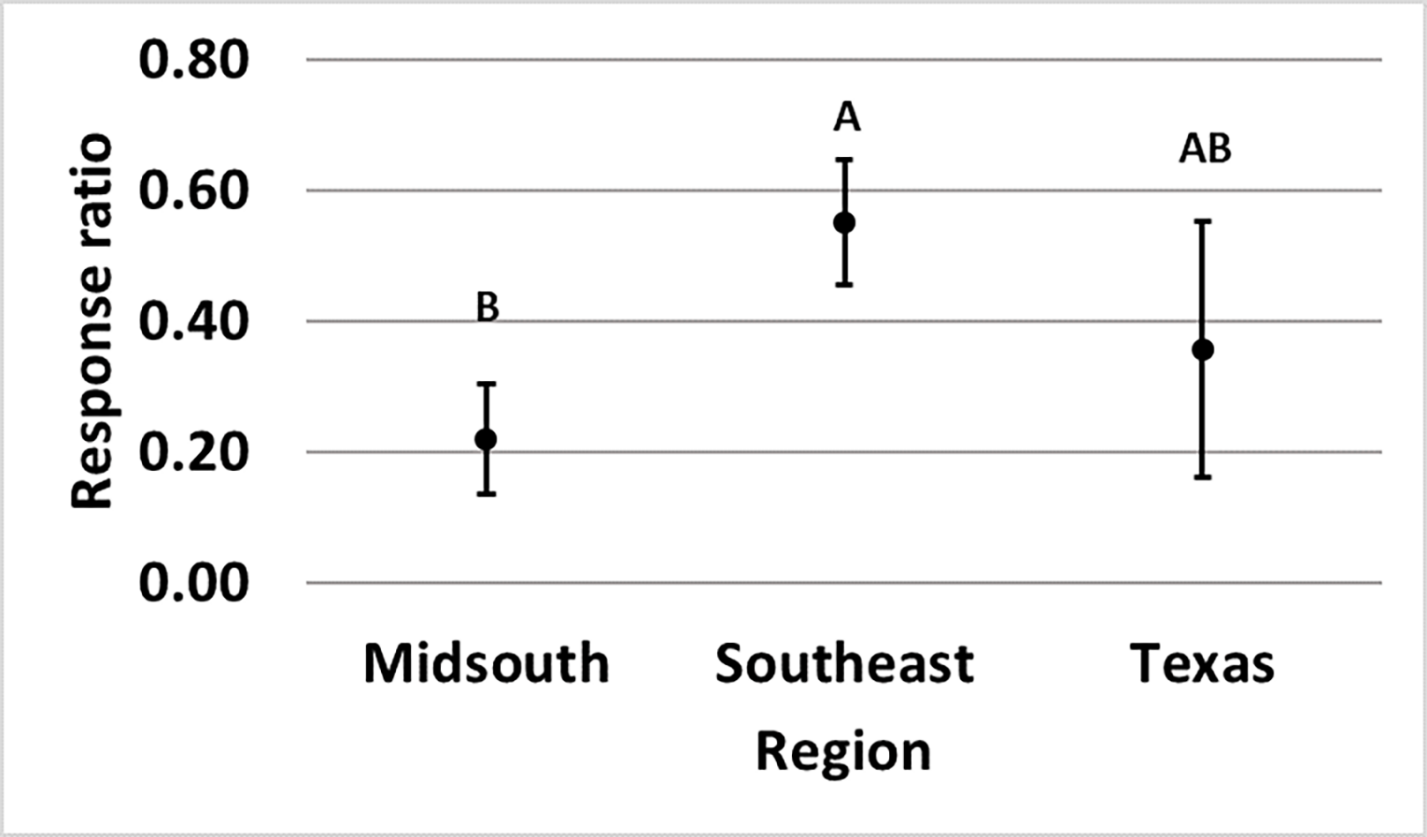
Least square mean ± SE of the response ratio of region of Bollgard cotton yield in trials from the eastern and central Cotton Belt of the United States. Regions not sharing the same uppercase letter are different (Least square means α = 0.05). Response ratio = ln ([Bollgard mean_x_ + 1] / [non-*Bt* mean_x_ + 1]).

The main effects and interaction of year and region were evaluated to determine if changes in yield of *Bt* technologies occurred over time or if yield was affected by region (S6 Table). There was an interaction of year and region for Bollgard II (Fig 11). The yield benefit over non-*Bt* cotton initially increased in both regions, but then began to decline beginning around 2010. This is consistent with the increased heliothine counts and damage observed in the Midsouth. WideStrike yields followed a similar trend (Fig 12). Region influenced Bollgard yield relative to non-*Bt* cotton (p = 0.0415). Yield increase of Bollgard was greater in the Southeast (73%) than in the Midsouth (25%) (Fig 13).

### Effects of plant part and region on *Bt* technologies

**Fig 14:**
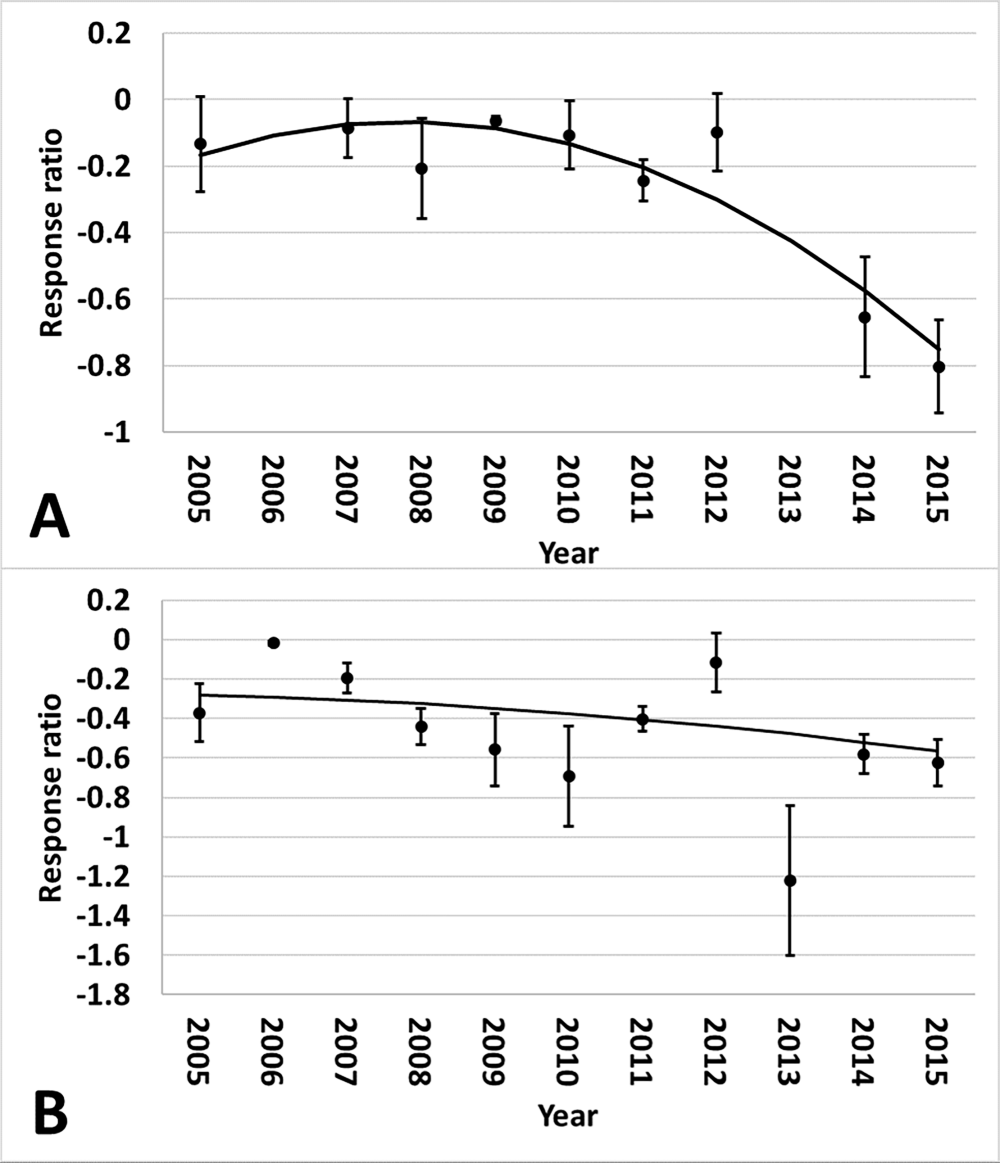
**Change over time of heliothine counts (A) and damage (B) of the comparison of Bollgard 2: WideStrike cotton in trials from the eastern and central Cotton Belt of the United States.** Heliothine counts equation: 0.3345x - 0.01305x2–2.2011, Damage equation: -0.0303x - 0.0581. Response ratio (A and B) = ln ([Bollgard II mean_x_ + 1] / [WideStrike mean_x_ + 1]).

**Fig 15:**
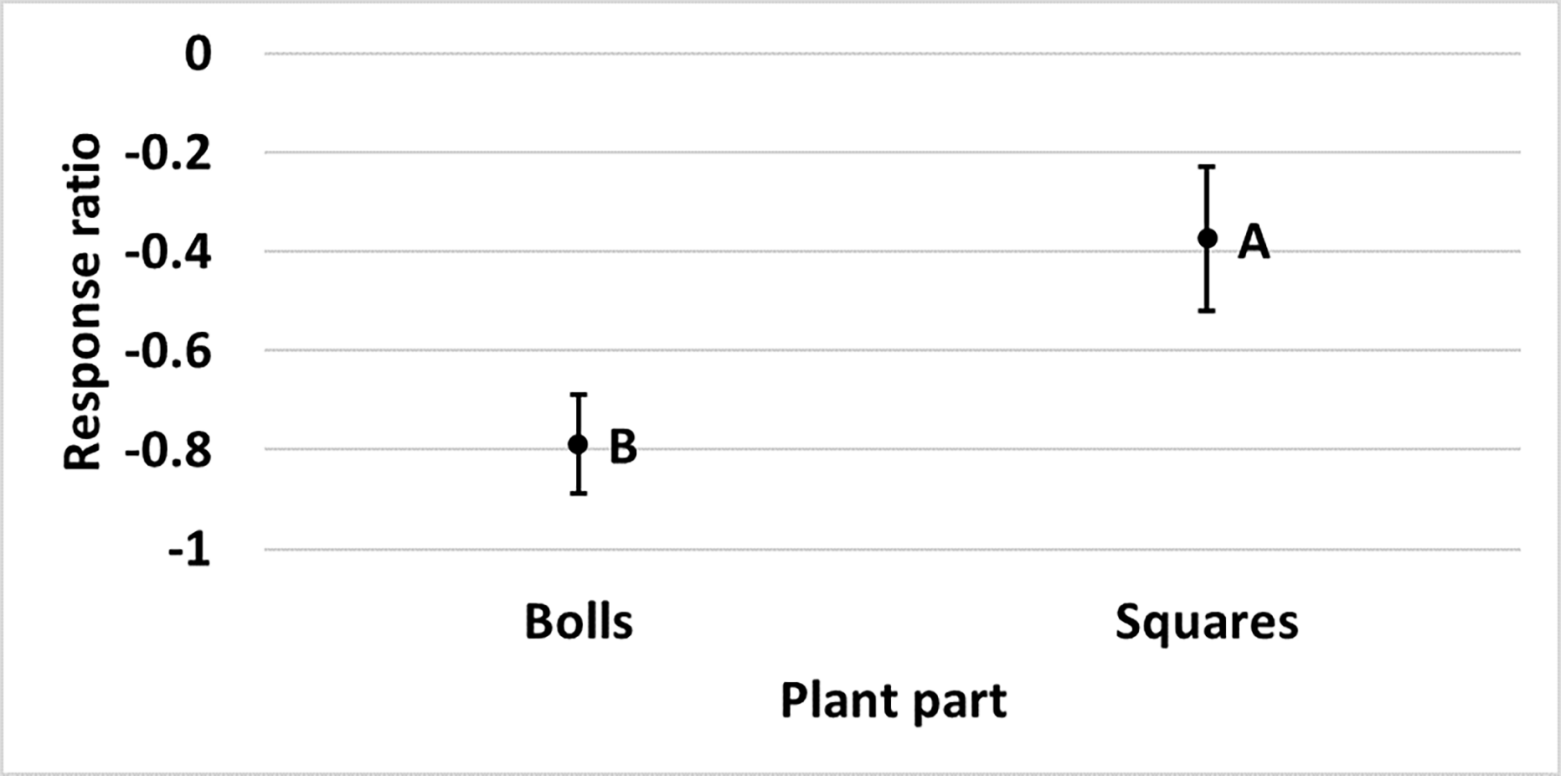
Least square mean ± SE of the response ratio of plant part of the comparison of Bollgard II: Bollgard damage in trials from the eastern and central Cotton Belt of the United States. Plant parts not sharing the same uppercase letter are different (Least square means α = 0.05). Response ratio = ln ([Bollgard II mean_x_ + 1] / [Bollgard mean_x_ + 1]).

**Fig 16:**
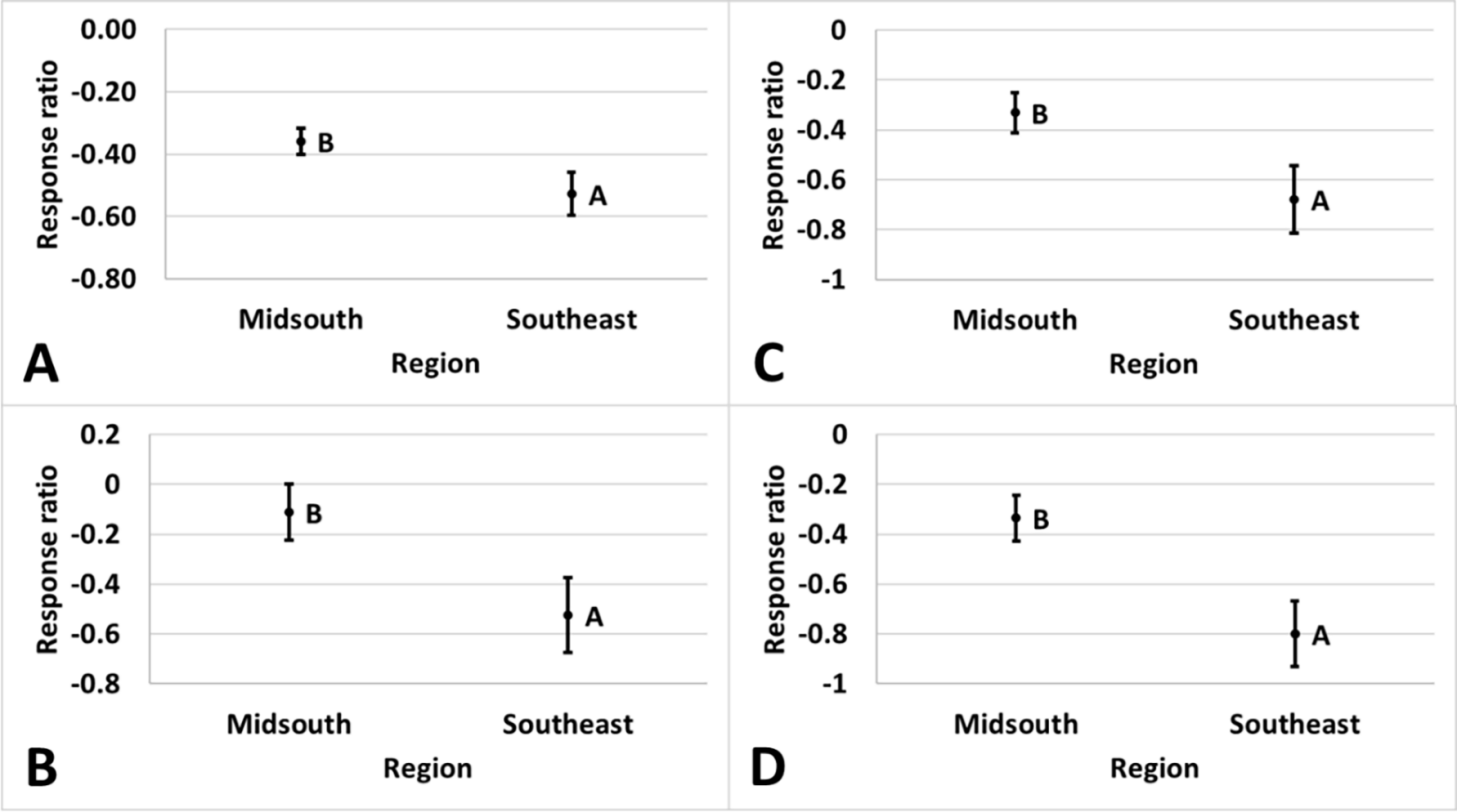
**Least square mean ± SE of the response ratio of damage by region of (A) Bollgard II: WideStrike, (B) Bollgard II: WideStrike 3, (C) TwinLink: WideStrike, and (D) WideStrike 3: WideStrike comparisons in trials from the eastern and central Cotton Belt of the United States.** Regions not sharing the same uppercase letter are different (Least square means α = 0.05). Response ratio (A) = ln ([Bollgard II mean_x_ + 1] / [WideStrike mean_x_ + 1]); Response ratio (B) = ln ([Bollgard II mean_x_ + 1] / [WideStrike 3 mean_x_ + 1]); Response ratio (C) = ln ([TwinLink mean_x_ + 1] / [WideStrike mean_x_ + 1]); Response ratio (D) = ln ([WideStrike 3 mean_x_ + 1] / [WideStrike mean_x_ + 1]).

**Fig 17:**
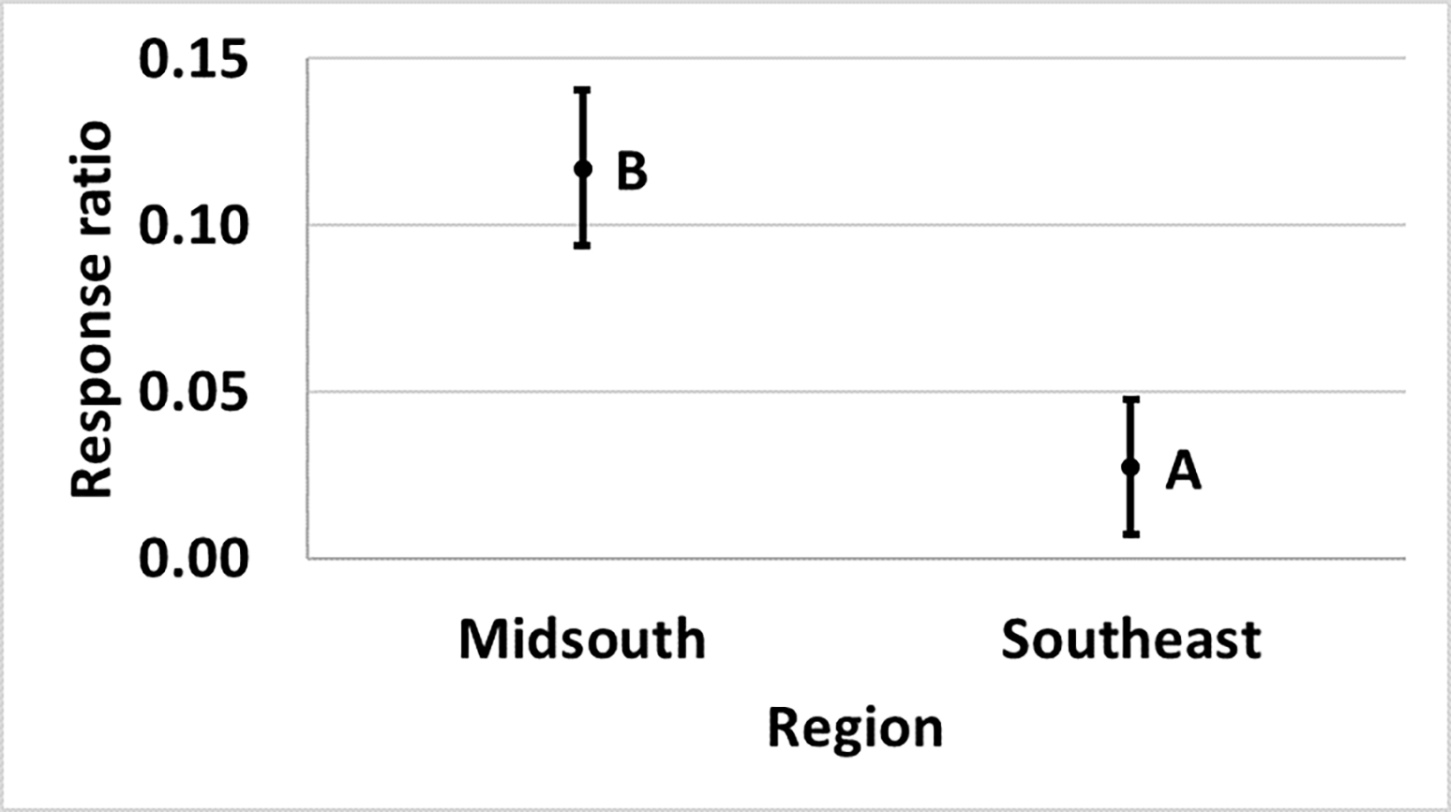
Least square mean ± SE of the response ratio of damage by region of the Bollgard II: WideStrike comparison in trials from the eastern and central Cotton Belt of the United States. Regions not sharing the same uppercase letter are different (Least square means α = 0.05). Response ratio = ln ([Bollgard II mean_x_ + 1] / [WideStrike mean_x_ + 1]).

The main effects of year, plant part, and region were evaluated to compare heliothine counts, damage and yield between *Bt* technologies (S6 Table). Year influenced heliothine counts (p<0.01) and damage (p = 0.03) in the Bollgard II: WideStrike comparison. Over time, the difference between Bollgard II and WideStrike increased for both heliothine counts and damage (Fig 14) as efficacy declined more rapidly in Widestrike than in Bollgard II. Plant part influenced the damage difference observed between Bollgard II and Bollgard. Damage reduction by Bollgard II compared to Bollgard was 54% on bolls and 31% on squares (Fig 15). Relative performance of comparisons between different *Bt* technologies varied by region for damage. Damage was reduced by 41% in the Southeast and 30% in the Midsouth in Bollgard II compared to WideStrike (p = 0.039), by 41% in the Southeast and 11% in the Midsouth in Bollgard II compared to TwinLink (p = 0.036), by 49% in the Southeast and 28% in the Midsouth in TwinLink compared to WideStrike (p = 0.034), and 55% in the Southeast and 28% in the Midsouth in Widestrike 3 compared to WideStrike (p = 0.006) (Fig 16). Region influenced the Bollgard II: WideStrike comparison of yield with Bollgard II having a greater yield benefit (13%) in the Midsouth than in the Southeast (3%) relative to WideStrike (p = 0.006) (Fig 17).

## Discussion

### Literature review

This paper reviewed published literature from 20 years of commercialized use of *Bt* cotton technologies; however, only six refereed articles fit the criteria for use in this paper and these data all occurred within the first 7 years of *Bt* cotton commercialization in the USA. The remainder of the data were from non-refereed sources or were unpublished data from university entomologists. The review revealed that although a large body of field-based *Bt* research exists, most of the information has not been subjected to peer-review. The primary reason for this is that many *Bt* field trials are stand-alone experiments and would not be appropriate for peer-review publications but fit well into report style publications such as the Proceedings of the Beltwide Cotton Conferences, Arthropod Management Tests, or Extension Service bulletins. However, the scrutiny of genetically modified crops, including *Bt* technologies, is increasing, and having more refereed, field-validated data will become increasingly important.

Texas accounted for only 5 percent of the data in this analysis; however, approximately 50 percent of the United States cotton acreage is in Texas [1]. Heliothine severity in Texas is lower than in the Midsouth and Southeast [1] and *Bt* technologies have provided exceptional suppression of heliothines. Therefore, less research on *Bt* cotton efficacy has been conducted in this region.

### Bias

Analyses to evaluate bias were not conducted as part of this study. Two main sources of bias were considered; however, these two sources, publication bias and selective reporting due to industry sponsorship, could not be effectively evaluated because the vast majority of the data used were from non-refereed sources and were conducted by entomologists in industry or receiving industry funds in their public university positions. This was unavoidable due to the nature of this type of research being conducted almost exclusively by entomologists who receive funding through industry to conduct applied research trials with commercial products to develop grower recommendations. Based on our knowledge, only three papers [36, 49, 50] may have been conducted without any possibility of industry influence or bias. These papers contributed 8 of 246 (3%), 5 of 585 (0.9%), and 3 of 580 (0.5%) data points for Bollgard, Bollgard II and Widestrike, respectively. This study had the advantage of having a large body of data from many sources across a wide breadth of locations and years, so the impact of any individual’s bias is minimal.

### Impacts of *Bt* technology on insecticide usage, heliothine counts, cotton damage, and yield

Cotton production practices in the USA have been impacted by *Bt* technology (Fig 2). The number of foliar insecticide applications in all *Bt* cotton technologies relative to non-*Bt* varieties were lowered, reducing environmental impacts from insecticides. Foliar insecticides are still often necessary in *Bt* cotton production and may become more important if resistance to *Bt* toxins becomes frequent and widespread. Newer *Bt* cotton technologies (TwinLink and WideStrike 3) were as good as or better than earlier *Bt* technologies for control of lepidopteran pests, but their impact on insecticide use could not be evaluated in this study. In the absence of foliar insecticide applications, differences between *Bt* and non-*Bt* cotton for heliothine counts, damage and cotton yield were documented for all technologies. Heliothine densities and damage were reduced, and yields of all technologies except WideStrike 3 increased. The combination of decreased insecticide use, decreased heliothine damage, and increased yields has been a substantial benefit of *Bt* technology for growers and the environment.

### Efficacy comparisons between *Bt* technologies and non-*Bt* varieties

Regional differences were found for *Bt* efficacy as measured by heliothine counts, damage, and yield for all technologies except TwinLink. Generally, the impact of technologies was greater in the Southeast than in the Midsouth. Bollgard and Bollgard II were the only technologies that had differences in relative damage between plant parts, with both providing more protection of bolls and squares than flowers, which is consistent with previous research [51, 52].

### Efficacy comparisons between *Bt* technologies

Bollgard II, WideStrike 3, and TwinLink all provided better control of heliothines than WideStrike regarding damage, and WideStrike provided better control than the single-gene product, Bollgard. Among the multi-gene technologies, the lower efficacy of WideStrike was likely due to its reliance on Cry1Ac, which was the first *Bt* gene inserted into commercial cotton varieties and has had resistance documented in *H*. *zea* [21, 22, 24], and the lack of efficacy of Cry1F against *H*. *zea* [25]. There was not a difference between WideStrike 3 and either Bollgard II or TwinLink, which was unexpected due to the addition of the Vip3A gene [53]. Only one year of data was available for WideStrike 3 comparisons and more research is needed before drawing conclusions on the impact of this new toxin. Unlike *Bt* to non-*Bt* comparisons where the non-*Bt* variety was normally a close genetic relative of the *Bt* variety, genetic similarity is not expected between *Bt* technologies developed by different companies. Therefore, some of the differences in yield between *Bt* technologies may have been due to differences in yield potential of the germplasm rather than the impact of the *Bt* toxins. Differences between damage on plant parts were observed only between Bollgard and Bollgard II and were consistent with comparisons of these technologies to non-*Bt* varieties. Regional differences between technologies were numerous and followed the same trend as comparisons between *Bt* and non-*Bt* where differences in technologies were greater in the Southeast than in the Midsouth. Taken together, these data reveal that multi-gene technology was superior to single-gene technology, thus demonstrating the need for additional pyramiding of novel *Bt* genes. Also, while performance varied depending on location and the aspect of efficacy being measured, relative performance of the technologies to each other and to non-*Bt* varieties was reasonably consistent.

### Changes in efficacy and yield over time

Evaluations of heliothine counts and damage over time revealed no changes in Bollgard efficacy from 1996 to 2008; however, a loss of efficacy occurred for both Bollgard II and WideStrike from introduction until 2015 in the Midsouth region (Figs 11 and 12). These technologies rely on three of the oldest commercialized *Bt* toxins (Cry1Ac, Cry2Ab and Cry1F) and resistance to Cry1Ac and Cry2Ab toxins has been documented [20, 22, 24, 54, 55]. Another contributing factor to the apparent loss of efficacy could be a shift to a higher proportion of *H*. *zea* in the heliothine complex. *Heliothis virescens* is more susceptible to Cry1Ac than *H*. *zea* [24], and therefore has a lower survival rate in *Bt* cotton. With the widespread adoption to *Bt* crops, population suppression of *H*. *virescens* may have occurred, resulting in *H*. *zea* comprising a higher proportion of the heliothine complex in non-Bt cotton [56, 57], resulting in the apparent loss of efficacy in *Bt* cotton even without a change in susceptibility toward either pest. The decline in efficacy reported here supports anecdotal observations of many entomologists in the Midsouth, and highlights the need for additional technologies for *H*. *zea* control, and the need for continued development of new insecticides and management tactics for lepidopteran pest management in cotton. The reason efficacy in the Southeast had not deteriorated is unknown, but could be related to different landscape diversity reducing selection pressure, a different source population that has experienced less selection, or *H*. *virescens* comprising a larger proportion of the heliothine complex in the Southeast. Given the mobility of *H*. *zea* [58–60], resistance developed in one part of the USA can spread rapidly throughout the country, so regional differences are unlikely to persist with this insect.

Evaluations of yield revealed complex changes over time. In both the Midsouth and Southeast regions, yield differences between *Bt* technologies (Bollgard II and WideStrike) and non-*Bt* varieties initially increased after commercialization, suggesting improved genetics of the varieties containing *Bt* technologies. After about 2010, these yield differences started to decrease, which is consistent with the increasing damage trends for these technologies in the Midsouth. While damage prevention and yield benefits appear to have decreased, *Bt* technologies still provided some protection from lepidopteran pests through 2015 which resulted in some yield benefits.

### Summary

Reductions in insecticide usage occurred with *Bt* cotton, but foliar insecticides were still needed to manage heliothine pests in many cases. *Bt* cotton reduced losses to heliothines and improved yields relative to non-*Bt* varieties, but economic benefits of these changes were not evaluated. Declining yield benefits of *Bt* technologies from around 2010 to 2015 were observed in the Midsouth and Southeast for Bollgard II and WideStrike technologies. Possible reasons for this are a decline in efficacy or a decline in insect pressure. Pheromone trap catches of heliothines would suggest that there is high annual variability in population size, but there was not a consistent trend from 2010–2015 (unpublished data, FRM). A decline in efficacy of *Bt* cotton was observed in the Midsouth, but not in the Southeast. This decline in efficacy could be due to insects becoming resistant to one or more *Bt* toxins or other changes being made in cotton genetics that alter susceptibility to heliothines. Since non-transgenic heliothine resistance is not a known goal for cotton breeders, it is most likely that changes in efficacy were due to insects developing resistance to the commercialized *Bt* toxins. This study was not able to distinguish counts and damage between *H*. *virescens* and *H*. *zea*. Since the authors are not aware of any *H*. *virescens* survival on any *Bt* cotton, it is assumed that changes in efficacy are due to changes in *H*. *zea* susceptibility. Given the mobile nature of *H*. *zea*, the resistance that was most pronounced in the Midsouth by 2015 may spread throughout the range of *H*. *zea*. As resistance becomes more common, the need to introduce new *Bt* technologies and improve other means of managing heliothine pests in cotton will increase. Furthermore, since *Bt* corn and *Bt* cotton use many of the same *Bt* toxins and *H*. *zea* develops on both crops, resistance management strategies should take both crops into consideration.

## Supporting information

S1 Table List of data sources in this paper used to conduct a meta-analysis of *Bt* cotton technologies. (PDF)

S2 Table Summary of the number of *Bt*: Non-*Bt* comparisons by publication type, insecticide use criteria, region, and technology from articles and unpublished data of trials in the eastern and central Cotton Belt of the United States. ^1^Arthropod Management Tests; ^2^Proceedings of the Beltwide Cotton Conferences; ^3^Extension publication; ^4^Thesis or dissertation; ^5^No data reported from T/D; ^6^ No data reported for other technologies.
(PDF)

S3 Table Summary of the number of *Bt*: *Bt* comparisons by publication type, insecticide use criteria, region, and technology from articles and unpublished data of trials in the eastern and central Cotton Belt of the United States. ^1^Arthropod Management Tests; ^2^Proceedings of the Beltwide Cotton Conference; ^3^Extension publication; ^4^Thesis or dissertation; ^5^No data reported from T/D; ^6^ No data reported for other comparisons.
(PDF)

S4 Table Summary of the number of *Bt*: Non-*Bt* comparisons of heliothine counts and damage and cotton yield by region and technology in trials from the eastern and central Cotton Belt of the United States. ^1^Data reported as a combination of bolls, flowers, and/or squares; ^2^Data reported as a combination of reproductive structures and terminals; ^3^No data reported for other technologies.
(PDF)

S5 Table Summary of the number of *Bt*: *Bt* comparisons of heliothine counts and damage and cotton yield by region and technology in trials from the eastern and central Cotton Belt of the United States. ^1^Data reported as a combination of bolls, flowers, and/or squares; ^2^Data reported as a combination of reproductive structures and terminals; ^3^No data reported for other comparisons.
(PDF)

S6 Table Statistical results of comparisons of *Bt* cotton technologies for heliothine counts, damage, and cotton yield overall, by plant part, region, year, and their interactions. n/e = not estimated.
(PDF)

S1 Fig Prisma checklist. (PDF)
