# Supplementary material for: Effects of transgenic Bacillus thuringiensis cotton on insecticide use, heliothine counts, plant damage, and cotton yield: A meta-analysis, 1996-2015
Source: PLoS One. 2018 Jul 19;13(7):e0200131. doi: 10.1371/journal.pone.0200131 (PMC6053876; doi:10.1371/journal.pone.0200131)
Supplement: S1 Table — (PDF) [file pone.0200131.s001.pdf]

Technology					Year(s) of study	Author	State(s)	Source
Bollgard®	Bollgard® II	WideStrike®	TwinLink®	WideStrike® 3				
x		x			2002	Adamczyk et al.	MS	Proc. 2002 Beltwide Cotton Conf. 1567-1571
x	x				2000	Adamczyk et al.	MS	J. Econ. Entomol. 94(6): 1589-1593
x	x				2000	Adamczyk et al.	MS	Proc. 2001 Beltwide Cotton Conf. 835-836
x	x				2000-2001	Akin et al.	MS	Proc. 2002 Beltwide Cotton Conf.
	x	x			2002 and 2004-2005	Ali and Braxton	GA	Proc. 2006 Beltwide Cotton Conf. 1095-1099
x	x				1999	Allen et al.	AR	Proc. 2000 Beltwide Cotton Conf. 1093-1094
x					1996 and 1998	Allen et al.	AR	Arthropod Manage. Tests 25(1): M15
x	x				2006	Bacheler and Mott	NC	Arthropod Manage. Tests 32(1): F21
x	x				2000	Bacheler and Mott	NC	Arthropod Manage. Tests 26(1): M8
x	x				2002	Bacheler and Mott	NC	Arthropod Manage. Tests 28(1): F41a
x	x				2003	Bacheler and Mott	NC	Arthropod Manage. Tests 29(1): F42
x	x				2001	Bacheler and Mott	NC	Arthropod Manage. Tests 27(1): M5
x	x				2000	Bacheler and Mott	NC	Proc. 2003 Beltwide Cotton Conf. 1011-1014
x					1998	Bacheler and Mott	NC	Arthropod Manage. Tests 24(1): F50
		x			2003	Bacheler and Mott	NC	Proc. 2004 Beltwide Cotton Conf. 1365-1368

x	x	x			2005	Bacheler et al.	NC	Proc. 2006 Beltwide Cotton Conf. 1536-1540
x					1996	Benedict et al.	TX	Arthropod Manage. Tests 24(1): M19
x					1996	Benedict et al.	TX	Arthropod Manage. Tests 24(1): F52
x					1998-1999	Brickle et al.	SC	J. Econ. Entomol. 94(1): 86-92
x	x				2002	Burris et al.	LA	Arthropod Manage. Tests 28(1): M6
x					1996	Burris et al.	LA	Proc. 1997 Beltwide Cotton Conf. 867-870
x					1998	Capps et al.	AR	Proc. 1999 Beltwide Cotton Conf. 1239-1241
	x	x			2010	Carter et al.	SC	Proc. 2011 Beltwide Cotton Conf. 1031-1038
		x			2007	Castro	TX	Arthropod Manage. Tests 33(1): M1
	x	x	x	x	2007-2014	Catchot	MS	Unpublished
x	x				2000-2001	Chitkowski et al.	SC	J. Econ. Entomol. 96(3): 755-762
	x	x			2007	Cook et al.	MS	Proc. 2008 Beltwide Cotton Conf. 1065-1069
x					2001-2003	Gable	LA	Louisiana State University Thesis
	x	x			2005-2015	Gore	MS	Unpublished
x	x	x	x	x	2007-2015	Greene	SC	Unpublished
x	x	x			2006-2009	Greene and Robinson	SC	Proc. 2010 Beltwide Cotton Conf. 1297-1302
x	x				2000-2002	Hagerty et al.	SC	Environ. Entomol. 34(1): 105-114
x	x				2001-2002	Hagerty et al.	SC	Proc. 2003 Beltwide Cotton Conf. 1161-1162

x	x				2006	Hardke et al.	AR	Arthropod Manage. Tests 33(1): F23
x					2001-2002	Head et al.	AL, GA, SC	Environ. Entomol. 34(5): 1257-1266
x					2001	Hopkins et al.	AR	Proc. 2002 Beltwide Cotton Conf.
		x			2004	Huckaba et al.	GA, NC, SC, VA	Proc. 2005 Beltwide Cotton Conf. 1252-1259
		x			2001-2002	Huckaba et al.	AL, LA, NC	Proc. 2003 Beltwide Cotton Conf. 1293-1298
x	x	x			2004	Jackson et al.	NC	Proc. 2005 Beltwide Cotton Conf. 1373-1378
x	x				1999	Jackson et al.	NC	Proc. 2000 Beltwide Cotton Conf. 1048-1052
x	x				2000	Jackson et al.	NC	Proc. 2001 Beltwide Cotton Conf. 815-818
	x	x			2005	Jackson et al.	NC	Proc. 2006 Beltwide Cotton Conf. 1531-1535
x	x				2002	Johnson et al.	AR	AAES Research Series 507
x	x				2001	Johnson et al.	AR	Arthropod Manage. Tests 28(1): M10
x	x				2001-2002	Johnson et al.	AR	Proc. 2003 Beltwide Cotton Conf. 1478-1480
x					2001	Johnson et al.	AR	Arthropod Manage. Tests 28(1): F57
	x	x	x	x	2008-2015	Kerns	LA	Unpublished
	x	x	x	x	2014	Kerns et al.	AR, LA, MS, TN	Proc. 2015 Beltwide Cotton Conf. 819-829
x					2001	Layton et al.	MS	Proc. 2002 Beltwide Cotton Conf.
x					1998	Layton et al.	MS	Proc. 1999 Beltwide Cotton Conf. 942-945

x					1997	Layton et al.	MS	Proc. 1998 Beltwide Cotton Conf. 970-973
x					1996	Layton et al.	MS	Proc. 1997 Beltwide Cotton Conf. 861-863
x					1999	Layton et al.	MS	Proc. 2000 Beltwide Cotton Conf. 1037-1039
x					2000	Layton et al.	MS	Proc. 2001 Beltwide Cotton Conf. 847-849
x	x				2000	Lentz et al.	TN	Proc. 2001 Beltwide Cotton Conf. 849-850
x					1996	Leonard et al.	LA	Proc. 1997 Beltwide Cotton Conf. 863-867
x					1997	Leonard et al.	LA	Proc. 1998 Beltwide Cotton Conf. 967-971
x	x	x	x	x	2008-2015	Lorenz	AR	Unpublished
x	x				2000	Lorenz et al.	AR	Proc. 2001 Beltwide Cotton Conf. 1116-1117
		x			2006	Lorenz et al.	AR	AAES Research Series 552
x					2002	Lorenz et al.	AR	Proc. 2003 Beltwide Cotton Conf. 1470-1472
x	x	x			2006-2007	Malone and Herbert	VA	Proc. 2008 Beltwide Cotton Conf. 1035-1042
x					2000	McGriff et al.	GA	Proc. 2001 Beltwide Cotton Conf. 431-432
x					2002	McGriff et al.	GA	Proc. 2003 Beltwide Cotton Conf. 850-854
	x	x			2005	Micinski et al.	LA	Proc. 2006 Beltwide Cotton Conf. 1090-1094
	x				2014-2015	Musser	MS	Unpublished
	x	x			2011-2012	Orellana et al.	AR	Proc. 2014 Beltwide Cotton Conf. 842-845

x	x				2003-2004	Parajulee	TX	Unpublished
x					2002	Parker	TX	Texas Cooperative Extension
		x			2004	Parker	TX	Texas Cooperative Extension
		x			2004	Parker and Livingston	TX	Proc. 2005 Beltwide Cotton Conf. 1687-1693
x					1997-1998	Pietrantonio and Heinz	TX	Proc. 1999 Beltwide Cotton Conf. 945-948
x					1998-1999	Reed et al.	MS	Proc. 2001 Beltwide Cotton Conf. 1131-1132
	x	x	x	x	2011-2015	Reisig	NC	Unpublished
x					1996	Scott et al.	MS	Proc. 1997 Beltwide Cotton Conf. 892-896
x					1998-1999	Seward et al.	TN	Proc. 2000 Beltwide Cotton Conf. 1055-1058
		x			2003	Siebert et al.	AR, GA, LA, MO, MS, NC, SC	J. Econ. Entomol. 101(6): 1950-1959
		x			2006	Siebert et al.	AR, LA, NC	Proc. 2007 Beltwide Cotton Conf. 1114-1118
		x			2003-2004	Smith et al.	AL	Proc. 2005 Beltwide Cotton Conf. 1330-1335
x	x				2003	Smith et al.	AR	Proc. 2004 Beltwide Cotton Conf. 1852-1853
x					2002	Sparks et al.	AR	Proc. 2002 Beltwide Cotton Conf.
	x	x	x	x	2011-2015	Stewart	TN	Unpublished
x					1999	Stewart et al.	MS	Proc. 2000 Beltwide Cotton Conf. 1043-1049
	x	x	x	x	2014	Taillon et al.	AR	Proc. 2015 Beltwide Cotton Conf. 252-255

x	x	x		x	2003-2011	Taylor	VA	Unpublished
x	x				2004	Tritt	TN	Proc. 2005 Beltwide Cotton Conf. 1443-1445
x	x				2004	Tritt and Burcham	TN	Proc. 2004 Beltwide Cotton Conf. 1354-1357
x	x				2000	Turnipseed et al.	SC	Proc. 2001 Beltwide Cotton Conf. 1009
