# Supplementary material for: Effects of transgenic Bacillus thuringiensis cotton on insecticide use, heliothine counts, plant damage, and cotton yield: A meta-analysis, 1996-2015
Source: PLoS One. 2018 Jul 19;13(7):e0200131. doi: 10.1371/journal.pone.0200131 (PMC6053876; doi:10.1371/journal.pone.0200131)
Supplement: S3 Table — 1Arthropod Management Tests; 2Proceedings of the Beltwide Cotton Conference; 3Extension publication; 4Thesis or dissertation; 5No data reported from T/D; 6 No data reported for other comparisons. (PDF) [file pone.0200131.s003.pdf]

Insecticide usage	Blanket[5]					None						Threshold[5]				
	Publication Type															
Region and Comparison	AMT[1]	BWCC[2]	Ext. pub[3]	Refereed	Unpublished	AMT[1]	BWCC[2]	Ext. pub[3]	Refereed	T/D[4]	Unpublished	AMT[1]	BWCC[2]	Ext. pub[3]	Refereed	Unpublished
**Midsouth**																
Bollgard® and Bollgard® II	6	35	3	0	0	9	37	3	1	0	18	0	6	0	0	0
Bollgard® and WideStrike®	0	0	0	0	0	0	3	0	0	0	18	0	0	0	0	0
Bollgard® II and WideStrike®	0	22	3	0	128	0	29	3	0	0	159	0	0	3	0	0
Bollgard® II and WideStrike® 3	0	11	3	0	23	0	11	3	0	0	24	0	0	0	0	0
Bollgard® II and TwinLink®	0	11	3	0	23	0	11	3	0	0	37	0	0	0	0	0
WideStrike® and WideStrike® 3	0	11	3	0	33	0	11	11	0	0	70	0	0	0	0	0
WideStrike® and TwinLink®	0	11	3	0	26	0	11	3	0	0	28	0	0	0	0	0
WideStrike® 3 and TwinLink®	0	11	3	0	26	0	11	3	0	0	28	0	0	0	0	0
**Southeast**																
Bollgard® and Bollgard® II	16	10	0	0	0	10	31	0	24	0	11	0	2	0	0	0
Bollgard® and WideStrike®	0	2	0	0	0	0	20	0	0	0	6	0	0	0	0	2
Bollgard® II and WideStrike®	0	21	0	0	13	0	59	0	0	0	40	0	8	0	0	2
Bollgard® II and WideStrike® 3	0	0	0	0	8	0	0	0	0	0	15	0	0	0	0	0
Bollgard® 2 and TwinLink®	0	0	0	0	4	0	0	0	0	0	47	0	0	0	0	0
WideStrike® and WideStrike® 3	0	0	0	0	8	0	0	0	0	0	57	0	0	0	0	0
WideStrike® and TwinLink®	0	0	0	0	4	0	0	0	0	0	19	0	0	0	0	0
WideStrike® 3 and TwinLink®	0	0	0	0	4	0	0	0	0	0	9	0	0	0	0	0
**Texas[6]**																
Bollgard® and Bollgard® II	0	0	0	0	10	8	1	0	0	0	15	0	0	0	0	0
