# Supplementary material for: Effects of transgenic Bacillus thuringiensis cotton on insecticide use, heliothine counts, plant damage, and cotton yield: A meta-analysis, 1996-2015
Source: PLoS One. 2018 Jul 19;13(7):e0200131. doi: 10.1371/journal.pone.0200131 (PMC6053876; doi:10.1371/journal.pone.0200131)
Supplement: S4 Table — 1Data reported as a combination of bolls, flowers, and/or squares; 2Data reported as a combination of reproductive structures and terminals; 3No data reported for other technologies. (PDF) [file pone.0200131.s004.pdf]

Region and Technology	Counts						Damage						Yield
	Bolls	Flowers	Repro[1]	Squares	Terminals	Whole plant[2]	Bolls	Flowers	Repro[1]	Squares	Terminals	Whole plant[2]	
**Midsouth**													
**Bollgard®**	2	6	1	21	16	23	39	6	1	46	15	6	83
**Bollgard® II**	36	26	8	32	22	18	82	37	17	75	50	2	88
**WideStrike®**	28	22	10	30	20	19	66	36	17	68	48	3	79
**WideStrike® 3**	2	0	4	1	0	0	9	5	0	9	8	0	10
**TwinLink®**	3	0	4	1	0	0	12	6	12	13	11	0	25
**Southeast**													
**Bollgard®**	9	3	0	4	0	92	29	0	4	6	1	0	46
**Bollgard® II**	26	4	0	19	2	7	70	3	0	32	15	0	70
**WideStrike®**	17	6	0	11	4	10	55	11	0	28	18	0	63
**WideStrike® 3**	0	0	0	0	0	2	5	3	0	5	5	0	3
**TwinLink®**	4	0	0	4	0	0	7	1	0	7	3	0	6
**Texas[3]**													
**Bollgard®**	2	2	2	17	3	9	11	7	0	23	3	1	10
**Bollgard® II**	1	1	0	1	1	6	7	6	0	7	1	0	7
**WideStrike®**	2	2	0	2	2	0	2	0	0	2	2	0	2
