# Supplementary material for: Effects of transgenic Bacillus thuringiensis cotton on insecticide use, heliothine counts, plant damage, and cotton yield: A meta-analysis, 1996-2015
Source: PLoS One. 2018 Jul 19;13(7):e0200131. doi: 10.1371/journal.pone.0200131 (PMC6053876; doi:10.1371/journal.pone.0200131)
Supplement: S5 Table — 1Data reported as a combination of bolls, flowers, and/or squares; 2Data reported as a combination of reproductive structures and terminals; 3No data reported for other comparisons. (PDF) [file pone.0200131.s005.pdf]

| Region and Technology | Counts | | | | | | Damage | | | | | | Yield |
|---|---|---|---|---|---|---|---|---|---|---|---|---|---|
| | Bolls | Flowers | Repro[1] | Squares | Terminals | Whole plant[2] | Bolls | Flowers | Repro[1] | Squares | Terminals | Whole plant[2] | |
| **Midsouth** | | | | | | | | | | | | | |
| **Bollgard® and Bollgard® II** | 2 | 6 | 0 | 5 | 8 | 17 | 15 | 6 | 0 | 21 | 7 | 2 | 29 |
| **Bollgard® and WideStrike®** | 2 | 2 | 0 | 2 | 2 | 0 | 3 | 2 | 0 | 3 | 2 | 0 | 3 |
| **Bollgard® II and WideStrike®** | 27 | 21 | 4 | 26 | 18 | 14 | 46 | 29 | 17 | 48 | 39 | 1 | 57 |
| **Bollgard® II and WideStrike® 3** | 2 | 0 | 4 | 0 | 0 | 0 | 9 | 6 | 12 | 10 | 10 | 0 | 22 |
| **Bollgard® II and TwinLink®** | 3 | 0 | 6 | 1 | 0 | 0 | 12 | 6 | 12 | 13 | 10 | 0 | 25 |
| **WideStrike® and WideStrike® 3** | 2 | 1 | 10 | 3 | 1 | 1 | 21 | 13 | 12 | 24 | 18 | 0 | 33 |
| **WideStrike® and TwinLink®** | 2 | 0 | 4 | 0 | 0 | 0 | 11 | 6 | 12 | 12 | 11 | 0 | 24 |
| **WideStrike® 3 and TwinLink®** | 2 | 0 | 4 | 0 | 0 | 0 | 11 | 6 | 12 | 12 | 11 | 0 | 24 |
| **Southeast** | | | | | | | | | | | | | |
| **Bollgard® and Bollgard® II** | 8 | 3 | 0 | 3 | 0 | 19 | 25 | 0 | 4 | 4 | 0 | 0 | 40 |
| **Bollgard® and WideStrike®** | 1 | 0 | 0 | 3 | 0 | 2 | 9 | 0 | 0 | 5 | 1 | 0 | 9 |
| **Bollgard® II and WideStrike®** | 13 | 0 | 0 | 7 | 0 | 7 | 39 | 3 | 0 | 19 | 8 | 0 | 47 |
| **Bollgard® II and WideStrike® 3** | 0 | 0 | 0 | 0 | 0 | 0 | 6 | 1 | 0 | 5 | 5 | 0 | 6 |
| **Bollgard® II and TwinLink®** | 7 | 0 | 0 | 7 | 0 | 0 | 13 | 1 | 0 | 10 | 4 | 0 | 9 |
| **WideStrike® and WideStrike® 3** | 3 | 3 | 0 | 4 | 3 | 2 | 12 | 6 | 0 | 11 | 11 | 0 | 10 |
| **WideStrike® and TwinLink®** | 2 | 0 | 0 | 2 | 0 | 0 | 5 | 1 | 0 | 5 | 3 | 0 | 5 |
| **WideStrike® 3 and TwinLink®** | 0 | 0 | 0 | 0 | 0 | 0 | 3 | 1 | 0 | 3 | 3 | 0 | 3 |
| **Texas[6]** | | | | | | | | | | | | | |
| **Bollgard® and Bollgard® II** | 1 | 1 | 0 | 1 | 1 | 7 | 7 | 6 | 0 | 7 | 1 | 0 | 7 |
