# Supplementary material for: Effects of transgenic Bacillus thuringiensis cotton on insecticide use, heliothine counts, plant damage, and cotton yield: A meta-analysis, 1996-2015
Source: PLoS One. 2018 Jul 19;13(7):e0200131. doi: 10.1371/journal.pone.0200131 (PMC6053876; doi:10.1371/journal.pone.0200131)
Supplement: S6 Table — n/e = not estimated. (PDF) [file pone.0200131.s006.pdf]

Comparison[1]	Response type	Effect	df[2]	F/t	p	% change[3]
**Bollgard: non-*Bt***	**Counts**	**Overall intercept**	41	-6.50	<0.0001	49.0
		**Plant part**	3, 38	0.46	0.7123	
		**Region**	2, 39	0.45	0.6425	
		**Year**	2, 37	1.82	0.1764	
		**Year*plant part**	n/e	n/e	n/e	
		**Year*region**	n/e	n/e	n/e	
	**Damage**	**Overall intercept**	88	-16.55	<0.0001	70.2
		**Plant part**	3, 86	2.79	0.0454	
		**Bolls**				71.9
		**Flowers**				48.2
		**Squares**				74.7
		**Terminals**				61.7
		**Region**	2, 87	3.33	0.0404	
		**Midsouth**				64.6
		**Southeast**				75.4
		**Texas**				76.5
		**Year**	2, 83	0.40	0.6728	
		**Year*plant part**	2, 66	0.08	0.92	
		**Year*region**	2, 58	2.99	0.0579	
	**Yield**	**Overall intercept**	59	5.82	<0.0001	43.8
		**Region**	2, 57	3.37	0.0415	
		**Midsouth**				24.6
		**Southeast**				73.4
		**Texas**				43.0
		**Year**	2, 54	1.82	0.1717	
		**Year*region**	n/e	n/e	n/e	
**Bollgard II: non-*Bt***	**Counts**	**Overall intercept**	111	-12.94	<0.0001	61.8
		**Plant part**	3, 108	2.50	0.0633	
		**Region**	1, 106	11.16	0.0012	
		**Midsouth**				56.8
		**Southeast**				73.8
		**Year**	2, 108	12.31	<0.0001	
		**Year*plant part**	2, 65	0.95	0.3916	
		**Year*region**	2, 101	6.62	0.002	
	**Damage**	**Overall intercept**	249	-34.03	<0.0001	81.4
		**Plant part**	3, 246	3.27	0.022	
		**Bolls**				83.3
		**Flowers**				74.4
		**Squares**				82.7
		**Terminals**				77.1

		**Region**	2, 247	6.59	0.0016	
		**Midsouth**				78.9
		**Southeast**				85.5
		**Texas**				85.1
		**Year**	2, 247	3.29	0.0388	
		**Year*plant part**	2, 183	0.43	0.6511	
		**Year*region**	2, 233	6.45	0.0019	
	**Yield**	**Overall intercept**	97	9.03	<0.0001	59.9
		**Region**	1, 93	4.92	0.0289	
		**Midsouth**				48.3
		**Southeast**				90.4
		**Year**	2, 96	7.71	0.0008	
		**Year*region**	2, 84	4.48	0.0141	
**WideStrike: non-*Bt***	**Counts**	**Overall intercept**	97	-12.52	<0.0001	47.4
		**Plant part**	3, 94	1.38	0.2533	
		**Region**	2, 95	14.23	<0.0001	
		**Midsouth**				48.4
		**Southeast**				58.4
		**Texas**				7.8
		**Year**	2, 95	0.29	0.7468	
		**Year*plant part**	2, 52	1.11	0.3375	
		**Year*region**	2, 84	12.90	<0.0001	
	**Damage**	**Overall intercept**	240	-28.58	<0.0001	68.4
		**Plant part**	3, 237	0.55	0.6456	
		**Region**	2, 238	2.56	0.0795	
		**Year**	2, 238	4.02	0.0192	
		**Year*plant part**	6, 220	0.43	0.8608	
		**Year*region**	2, 229	7.09	0.001	
	**Yield**	**Overall intercept**	89	8.65	<0.0001	54.2
		**Region**	1, 86	2.28	0.1347	
		**Year**	2, 87	5.45	0.0059	
		**Year*region**	2, 79	0.70	0.5001	
**WideStrike 3: non-*Bt***	**Counts**	**Overall intercept**	n/e	n/e	n/e	
		**Plant part**	n/e	n/e	n/e	
		**Region**	n/e	n/e	n/e	
	**Damage**	**Overall intercept**	34	-8.80	<0.0001	79.7
		**Plant part**	3, 31	0.86	0.4736	
		**Region**	1, 33	8.32	0.0068	
		**Midsouth**				70.7
		**Southeast**				89.0
	**Yield**	**Overall intercept**	7	4.18	0.0041	23.0
		**Region**	n/e	n/e	n/e	

**TwinLink: non-*Bt***	**Counts**	**Overall intercept**	10	-6.62	<0.0001	69.3
		**Plant part**	1, 9	0.03	0.8615	
		**Region**	n/e	n/e	n/e	
	**Damage**	**Overall intercept**	37	-11.37	<0.0001	71.8
		**Plant part**	2, 31	0.71	0.5008	
		**Region**	1, 36	0.19	0.6623	
	**Yield**	**Overall intercept**	17	4.71	0.0002	64.7
		**Region**	1, 16	0.01	0.9348	
**Bollgard II: Bollgard**	**Counts**	**Overall intercept**	16	-1.82	0.087	
		**Plant part**	1, 9	1.68	0.2271	
		**Region**	1, 11	2.56	0.1379	
	**Damage**	**Overall intercept**	36	-8.21	<0.0001	46.7
		**Plant part**	1, 27	5.42	0.0277	
		**Bolls**				54.5
		**Squares**				31.2
		**Region**	2, 34	0.46	0.6366	
	**Yield**	**Overall intercept**	18	0.23	0.8215	
		**Region**	n/e	n/e	n/e	
**Widestrike: Bollgard**	**Counts**	**Overall intercept**	9	-1.87	0.095	
		**Plant part**	n/e	n/e	n/e	
		**Region**	n/e	n/e	n/e	
	**Damage**	**Overall intercept**	18	-2.70	0.0146	20.5
		**Plant part**	1, 12	0.24	0.6327	
		**Region**	1, 17	0.01	0.9132	
	**Yield**	**Overall intercept**	7	-0.47	0.6493	
		**Region**	n/e	n/e	n/e	
**Bollgard II: WideStrike**	**Counts**	**Overall intercept**	62	-4.92	<0.0001	17.9
		**Plant part**	3, 59	0.91	0.4431	
		**Region**	1, 61	0.22	0.6417	
		**Year**	2, 60	13.45	<0.0001	
		**Year*plant part**	n/e	n/e	n/e	
		**Year*region**	n/e	n/e	n/e	
	**Damage**	**Overall intercept**	141	-10.99	<0.0001	33.4
		**Plant part**	3, 138	0.76	0.5199	
		**Region**	1, 140	4.32	0.0394	
		**Midsouth**				30.2
		**Southeast**				41.0
		**Year**	2, 139	3.51	0.0326	
		**Year*plant part**	2, 93	0.74	0.4819	
		**Year*region**	2, 136	0.27	0.7611	
	**Yield**	**Overall intercept**	53	4.04	0.0002	6.8
		**Region**	1, 52	8.41	0.0055	

		**Midsouth**				12.4
		**Southeast**				2.8
		**Year**	2, 51	0.99	0.3787	
		**Year*region**	n/e	n/e	n/e	
**WideStrike 3: Bollgard II**	**Counts**	**Overall intercept**	n/e	n/e	n/e	
		**Plant part**	n/e	n/e	n/e	
		**Region**	n/e	n/e	n/e	
	**Damage**	**Overall intercept**	16	1.12	0.2792	
		**Plant part**	2, 12	0.10	0.9036	
		**Region**	1, 15	0.00	0.9494	
	**Yield**	**Overall intercept**	5	2.89	0.034	12.7
		**Region**	n/e	n/e	n/e	
**Bollgard II: TwinLink**	**Counts**	**Overall intercept**	10	-3.73	0.0039	38.2
		**Plant part**	1, 9	0.15	0.709	
		**Region**	n/e	n/e	n/e	
	**Damage**	**Overall intercept**	32	-2.73	0.0101	23.0
		**Plant part**	2, 27	2.25	0.1252	
		**Region**	1, 31	4.79	0.0363	
		**Midsouth**				10.6
		**Southeast**				40.8
	**Yield**	**Overall intercept**	16	-0.65	0.5262	
		**Region**	1, 15	0.00	0.946	
**TwinLink: WideStrike**	**Counts**	**Overall intercept**	4	2.51	0.0658	
		**Plant part**	n/e	n/e	n/e	
		**Region**	n/e	n/e	n/e	
	**Damage**	**Overall intercept**	31	5.77	<0.0001	34.5
		**Plant part**	2, 25	1.52	0.2386	
		**Region**	1, 30	4.92	0.0342	
		**Midsouth**				28.2
		**Southeast**				49.3
	**Yield**	**Overall intercept**	15	-4.64	0.0003	11.8
		**Region**	n/e	n/e	n/e	
**WideStrike 3: WideStrike**	**Counts**	**Overall intercept**	n/e	n/e	n/e	
		**Plant part**	n/e	n/e	n/e	
		**Region**	n/e	n/e	n/e	
	**Damage**	**Overall intercept**	34	5.93	<0.0001	38.6
		**Plant part**	3, 31	0.92	0.4445	
		**Region**	1, 33	8.59	0.0061	
		**Midsouth**				28.5
		**Southeast**				55.1
	**Yield**	**Overall intercept**	7	-0.28	0.7903	
		**Region**	n/e	n/e	n/e	

**WideStrike 3: TwinLink**	**Counts**	**Overall intercept**	n/e	n/e	n/e	
		**Plant part**	n/e	n/e	n/e	
		**Region**	n/e	n/e	n/e	
	**Damage**	**Overall intercept**	15	1.12	0.2783	
		**Plant part**	1, 8	0.23	0.6431	
		**Region**	1, 14	0.02	0.8768	
	**Yield**	**Overall intercept**	5	4.67	0.0055	8.2
		**Region**	n/e	n/e	n/e	

[1]Technology$_1$: Technology$_2$

[2]Where df only displays one value, it is the denominator degrees of freedom and the test was a t-test.

[3]Percent reduction of counts or damage or percent increase of yield by Technology$_1$ vs. Technology$_2$ for significant comparisons not including year effects for regression analyses.

n/e= data was not estimatable due to a lack of available comparisons.
